# Colposcopic Imaging Carries Most of the Signal: Externally Validated Tri-Modal Integration of Imaging, Vaginal Microbiome, and Coagulation-Immune Data in HPV-Associated Co-Infection

**DOI:** 10.3390/diagnostics16182992

**Published:** 2026-09-16

**Authors:** Jinyi Zhang, Li Shu

**Affiliations:** Department of Gynecology, The Second Affiliated Hospital and Yuying Children’s Hospital of Wenzhou Medical University, 109 Xueyuan West Road, Lucheng District, Wenzhou 325000, China; zhangjinyi@wzhealth.com

**Keywords:** colposcopy, multimodal deep learning, vaginal microbiome, D-dimer-to-lymphocyte ratio, human papillomavirus, genital co-infection, external validation

## Abstract

**Background/Objectives:** To determine whether a coagulation-immune index, vaginal microbiome composition, and colposcopic imaging carry non-redundant information about HPV-associated lower genital tract co-infection when integrated within a single learned model. **Methods:** This retrospective three-center study analyzed 560 women referred for colposcopy: 420 formed the development cohort, and 140 were a withheld external cohort from a third center. All three modalities were sampled within one visit. Participants were classified as HPV-negative, HPV-positive without co-infection, or HPV-positive with co-infection, the latter confirmed by nucleic acid amplification for Chlamydia trachomatis, Neisseria gonorrhoeae, Trichomonas vaginalis, or Mycoplasma genitalium, with overlapping taxa masked a priori. TIM-Fusion combined a ConvNeXt-Tiny image encoder, a feature-tokenizer transformer for the D-dimer-to-lymphocyte ratio, and a perceptron for centered log-ratio abundances through modality-confidence weighting and bottleneck cross-attention. **Results:** On the external cohort, TIM-Fusion achieved a macro-AUC of 0.92 (95% CI 0.86 to 0.96) and accuracy of 0.90 across the three categories, exceeding the image-only baseline at 0.81, the tabular-only baseline at 0.76, and the best pairwise combination at 0.86. All thirteen comparisons, differences of 0.03 to 0.16, retained significance under Holm correction. Ablation attributed 0.05 (0.01 to 0.10) to bottleneck fusion, the only component with an interval excluding zero; withholding imaging, microbiome, or the ratio cost 0.13, 0.06, and 0.04. Imaging was therefore the dominant contributor, its removal costing three times as much as either of the other modalities. Entering D-dimer and lymphocyte count separately rather than as a ratio gave 0.89 (0.82 to 0.93), so the ratio form is an estimation convenience at this sample size and not a requirement. Co-infection-class AUC was 0.87 (0.75 to 0.94) on 26 events. In a secondary analysis restricted to the 84 HPV-positive external participants, binary discrimination of co-infection was 0.86 (0.76 to 0.93) with sensitivity 0.85 and specificity 0.91, lower than the one-versus-rest figure, which includes HPV-negative women in the negative class. **Conclusions:** Colposcopic morphology carries most of the discriminatory signal, while vaginal microbial ecology and coagulation-immune status each add information the others do not. Discrimination of co-infection status itself is more modest than the macro-averaged figure suggests, since within the HPV-positive stratum alone it falls to 0.86 on 26 events.

## 1. Introduction

Cervical cancer remains one of the most common malignancies affecting women worldwide, with an estimated 661,000 new cases and 348,000 deaths annually and a burden projected to rise absent accelerated intervention [1]. Persistent infection with high-risk HPV genotypes is its principal necessary cause, and the World Health Organization has set 90-70-90 targets for vaccination, screening, and treatment coverage, respectively, though most countries remain off track [2]. The trajectory from acquisition to persistent infection and lesion progression is nonetheless shaped by host and microenvironmental factors beyond viral presence, and concurrent infection of the lower genital tract is increasingly recognized as one such factor: Latorre-Millán et al. [3] found co-infection with other sexually transmitted pathogens more frequent among women with persistent high-risk HPV infection, supporting co-infection status as a clinically meaningful stratum rather than an incidental finding.

Two largely separate strands of biomarker research show that host physiological state carries signal for cervical disease independent of direct viral detection. The first concerns systemic coagulation and immune activation, in which coagulation-cascade activation mediated through tissue factor expression and platelet-fibrin-tumor cell interaction underlies the rationale for markers such as D-dimer [4]. Zhuang et al. [5] synthesized 38 cohort studies comprising 10,246 patients and confirmed that pretreatment neutrophil-to-lymphocyte ratio predicts survival, while Chen Q et al. [6], in 888 patients, found elevated D-dimer independently predicted postoperative recurrence in early-stage disease (adjusted OR 2.16, 95% CI 1.28 to 3.62). The second concerns the local vaginal environment, where a shift away from Lactobacillus-dominant communities toward a diverse, dysbiotic state correlates with HPV persistence and lesion severity [7,8].

Throughout that literature, coagulation and immune-cell indices are treated as standalone variables evaluated against a single endpoint by cutoff, hazard ratio, or odds ratio. The reframing proposed here concerns that role rather than the algebra of the index: the D-dimer-to-lymphocyte ratio (DLR) is treated not as an isolated marker read against a threshold, but as one encoded input stream within a network that learns how it interacts with two other biological compartments. The ratio form rests on the narrower hypothesis that D-dimer and absolute lymphocyte count capture two limbs of the same host response moving in opposite directions under sustained mucosal inflammation, so that their quotient attenuates between-individual variation while retaining the direction of the joint signal. This is a hypothesis about convenience of parameterization rather than about information content, since the quotient is a deterministic function of its components and cannot contain anything they do not, so the parameterization is tested empirically in Section 3.4 rather than assumed.

The index is described as coagulation-immune rather than as a marker of thromboinflammation, since D-dimer lies predominantly within the reference range here, no other marker of coagulation activation was measured, and the stronger term would assert a mechanism these data cannot support. Because the supporting evidence derives almost entirely from patients with established carcinoma, whether such an index retains value earlier in the disease spectrum is untested, and the branch’s contribution can be quantified against two modalities that sample the affected compartment directly.

Deep learning has meanwhile been applied extensively to colposcopic imaging [9], motivated by the variability and training burden of expert interpretation, with meta-analytic estimates indicating clinically acceptable discrimination and continuing calls for external validation before translation [10]. Aquilina and Papagiannakis [11] showed that a classifier on images alone achieved moderate discrimination but that fusing image-based predictions with structured clinical and referral data materially improved it. Madathil et al. [12] found transformer-based fusion best among five strategies on internal validation, which motivated the attention-based fusion adopted here [13], and Himabindu et al. [14] reported high internal-validation accuracy from a Swin-transformer ensemble; all three are reimplemented as comparators, each in its original two-modality form and a three-modality extension.

No study identified by the search strategy applied here has combined all three modalities within a single learned model, and the imaging and multimodal studies identified relied on internal rather than fully independent external validation. This study develops such a model, TIM-Fusion, and uses it as an instrument to ask whether the three compartments carry non-redundant information, with four objectives: to test whether combining them improves classification over any single modality or pairwise combination; to compare the architecture against published fusion paradigms reimplemented under an identical protocol with identical inputs; to attribute any gain to specific architectural components; and to characterize calibration, subgroup consistency, and interpretability. A fifth question, added during revision and therefore secondary and post hoc, asks how well the model separates co-infection within the HPV-positive stratum alone, which is the contrast of most direct clinical interest and the one the three-class formulation reports only indirectly. The aim is not a clinical tool. The scale of the experiments follows from that aim, since attributing information to individual modalities and architectural components under multiplicity control requires many more fits than running a model would. Because the outcome is a laboratory result concurrent with the model inputs, the task is diagnostic rather than prognostic, and the same panel that defines the outcome would in practice answer the question the model addresses. The same holds for HPV status, which is determined by genotyping already performed in this referral pathway, so the three-category structure is adopted to keep the co-infection contrast in its native population rather than to propose prediction of viral status. What the analysis can establish is how the three compartments relate, not whether a model built on them should be used.

## 2. Materials and Methods

### 2.1. Study Design and Ethical Approval

This was a retrospective multicenter cohort study across three independent gynecologic and pathology centers, using consecutively identified records of women referred for colposcopic examination. The protocol was approved by the ethics committee of Wenzhou Medical University, with a waiver of informed consent granted because only routinely collected clinical data were used, and specific written consent for publication was obtained for every participant whose image appears in a figure. All identifiers were removed before analysis, and each participant was assigned a de-identified code used consistently across the three modalities.

### 2.2. Study Population and Eligibility

**Figure 1:**
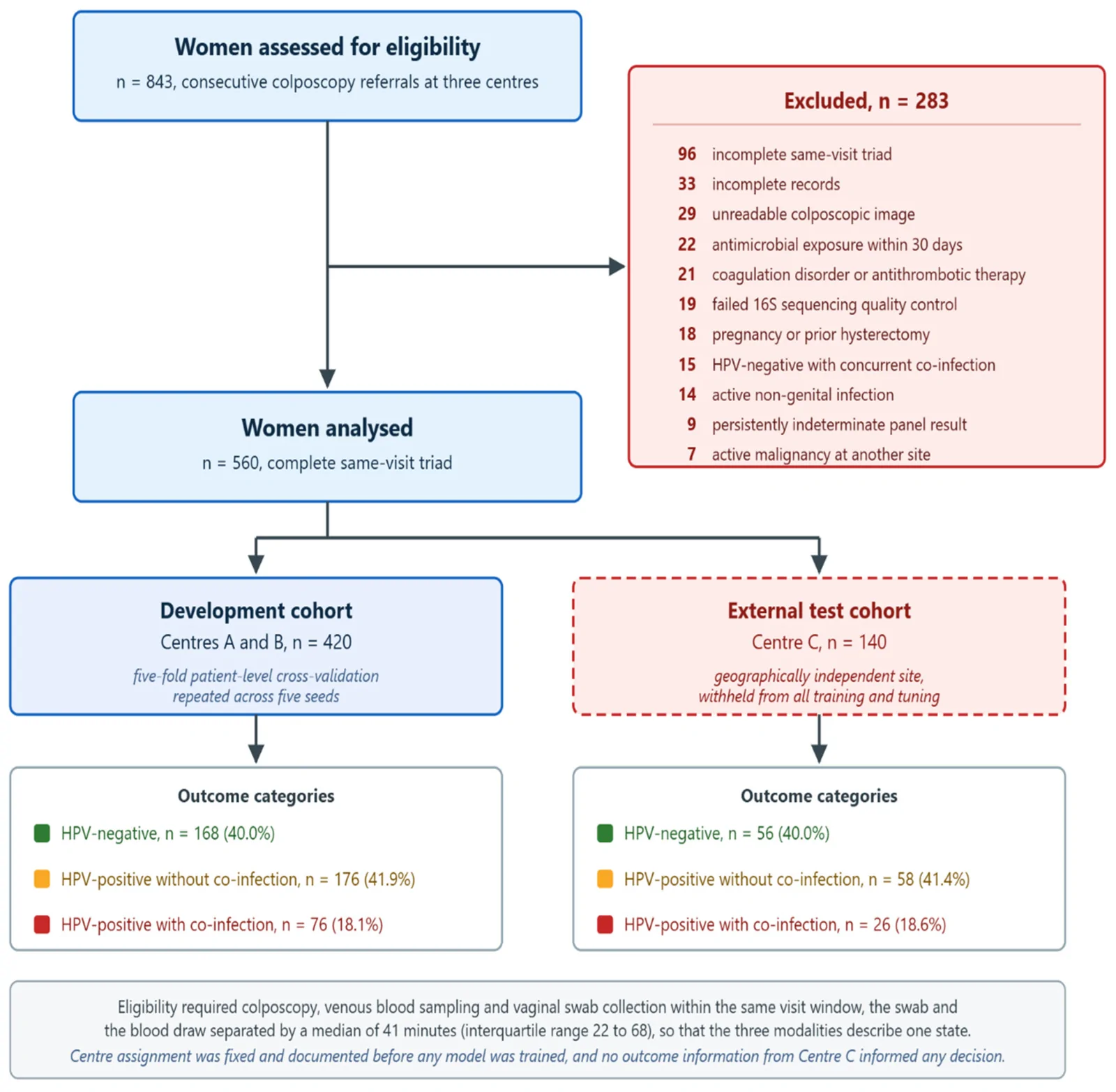
Participant flow.

Participants entered through a colposcopy referral pathway rather than primary screening, so the case mix is not that of an unselected screening population, and their flow from eligibility assessment to analysis is given in Figure 1. Referral indication, cytology grade, and histopathological lesion grade were recorded for every participant and are reported with the cohort description below. Table 1 lists the eligibility and exclusion criteria applied.

Eligibility required age 18 to 65 years with colposcopy, venous sampling, and vaginal swabbing within one visit window, the two draws separated by a median of 41 min (IQR 22 to 68) so that the three modalities describe the same state, plus a coagulation panel within 24 h and one interpretable transformation-zone image. Exclusions, listed in Table 1, covered conditions altering coagulation and immune-cell markers independently of genital tract status, antimicrobial exposure within 30 days, technical failures, and HPV-negative women with co-infection, whom the three-category outcome cannot accommodate.

### 2.3. Sample Size

Development sample size followed Riley et al. [15]. Assuming a C-statistic of 0.85, smallest-category prevalence 0.18, shrinkage 0.90, and 18 free parameters, the minimum was 391, met by the achieved 420. It is orienting only: the criterion assumes countable regression parameters, and effective degrees of freedom are undefined for a partially fine-tuned network. Regularization was therefore structural: the imaging backbone was frozen except for its final stage, the microbiome vector was compressed to 40 dimensions, and dropout was applied at one fusion representation.

External validation criteria [16] recommend at least 100 events and 100 non-events for a precise calibration slope; the external cohort of 140 contains at most 58 events per category and 26 with co-infection, so class-specific estimates are imprecise.

### 2.4. Multi-Center Data Partitioning

**Figure 2:**
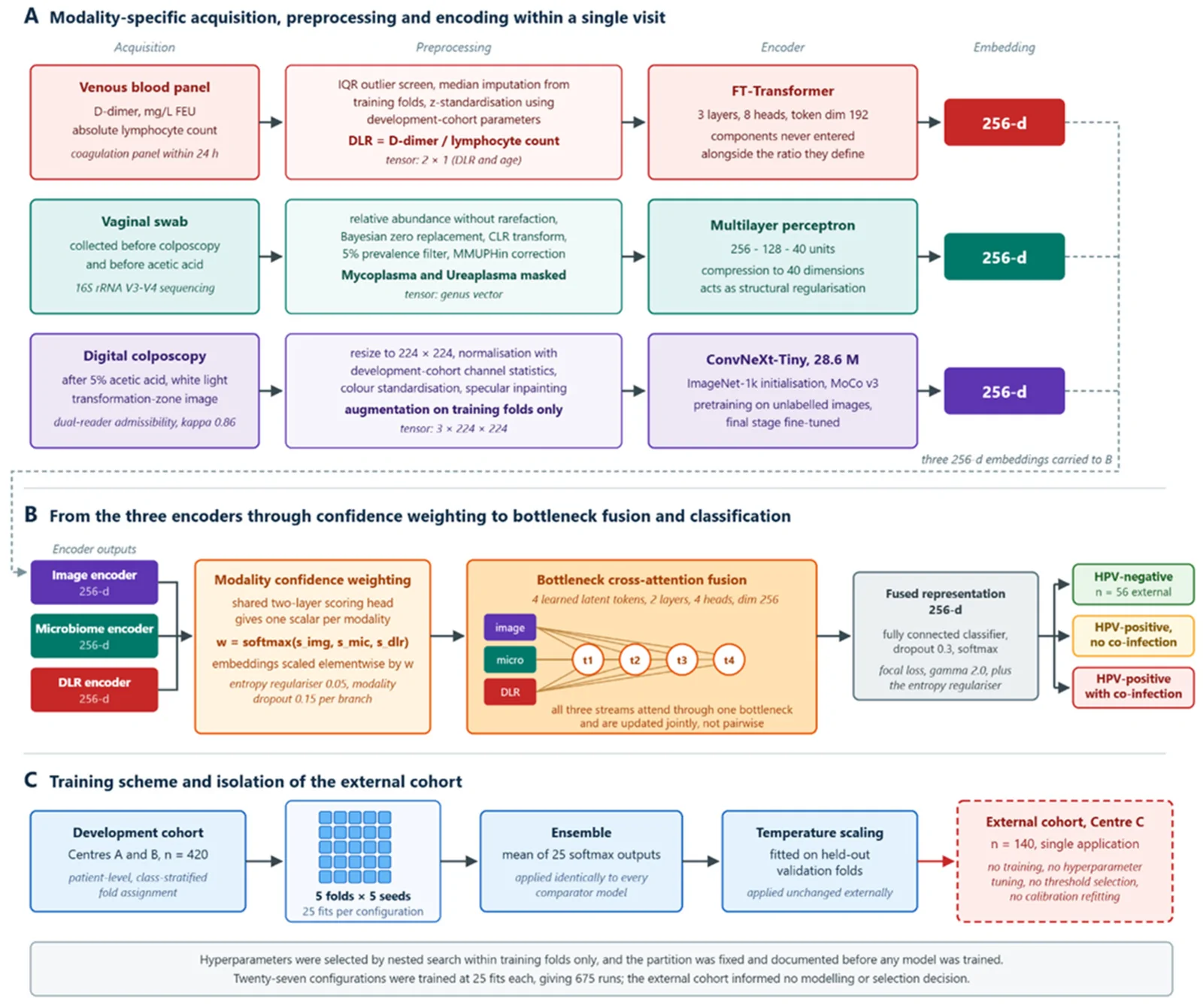
Study workflow and model architecture.

Two centers were pooled for development, with training and internal validation by five-fold patient-level cross-validation ensuring no patient contributed to more than one fold, while the third, geographically and institutionally independent center was withheld entirely as an external test set. That assignment was fixed and documented before any model was trained, and no outcome information from it informed any modelling or hyperparameter decision. Centers are denoted A and B for development and C for external testing. Colposcopy units, sequencing platforms, and coagulation assays differed between sites; D-dimer results were harmonized to fibrinogen-equivalent units, and the DLR is expressed as mg/L FEU per 10^9^ lymphocytes/L. Figure 2 sets out the workflow from acquisition to external validation.

### 2.5. Data Modalities and Acquisition

Venous sampling yielded D-dimer concentration and absolute lymphocyte count, from which the DLR was derived; platelet count, fibrinogen, and C-reactive protein were also recorded, the last two inconsistently across sites, and none was entered into the model. A vaginal swab was collected before colposcopy and before acetic acid application, followed by 16S rRNA sequencing of the V3 to V4 region, with reads resolved to amplicon sequence variants and classified against a curated cervicovaginal reference permitting species-level assignment of Lactobacillus [17], on which community state type was assigned; where assignment was ambiguous, the sample was categorized only as dominant or non-dominant. Colposcopic images were acquired after 5% acetic acid using digital colposcopy units under white light at a fixed working distance.

### 2.6. Data Preprocessing

Tabular: Outliers were screened by the interquartile range method. Missingness was 2.1% (D-dimer) and 0.9% (absolute lymphocyte count) for the two modelled variables, and 0.7% (platelet count), 14.3% (fibrinogen), and 18.6% (C-reactive protein). Missing values took the training-fold median, and externally the development-cohort median, with a complete-case sensitivity analysis below; continuous variables were z-standardized on development-cohort parameters.

Microbiome: Counts became relative abundances without rarefaction, which discards information in compositional data. Zeros received Bayesian-multiplicative replacement before centered log-ratio transformation, and taxa in fewer than 5% of samples were filtered out. The prevalence filter, geometric mean, and scaling constants came from the development cohort alone.

Imaging: Images were resized to 224 by 224 pixels and normalized on development-cohort channel statistics, with color standardization for inter-center illumination and camera differences and inpainting of specular reflection.

Batch effects: Microbiome data were harmonized across centers using MMUPHin with center as the batch variable [18], and Center C parameters estimated from its own unlabeled sequencing output. This uses no outcome information and places training and testing in one input space. Being transductive, harmonization requires a batch of samples rather than a single patient, which no prospective deployment on individual women could supply. The uncorrected results reported in Section 3.9 are therefore the deployment-relevant estimates, and the harmonized results reported elsewhere describe performance when a full external batch is available at once, as it was here.

### 2.7. Outcome Definition and Separation of Input from Outcome

Co-infection was laboratory-confirmed detection of Chlamydia trachomatis, Neisseria gonorrhoeae, Trichomonas vaginalis, or Mycoplasma genitalium by nucleic acid amplification, with per-organism counts reported per cohort. Participants were assigned to three categories: HPV-negative, HPV-positive without co-infection, or HPV-positive with co-infection; HPV status was determined by polymerase chain reaction genotyping that also separated HPV16 and HPV18. The three-category formulation is an analytical device, not a proposal to predict HPV status. Genotyping is available before colposcopy in this pathway, and no model output should substitute for it. Retaining HPV-negative women serves two purposes. It keeps the class structure of the population the model would encounter, so that class-specific estimates are not made in an artificially restricted stratum, and it provides a specificity check on the imaging branch, since a model that had learned only lesion appearance would be expected to confuse HPV-negative women with HPV-positive women without co-infection rather than to separate them. Section Secondary Analysis: Co-Infection Within the HPV-Positive Stratum reports the contrast of clinical interest, co-infection within the HPV-positive stratum, without the HPV-negative participants in the negative class. Because 16S is also an input, the panel was restricted to organisms that V3 to V4 sequencing captures poorly; bacterial vaginosis was excluded; Mycoplasma and Ureaplasma were masked a priori, and a prespecified sensitivity analysis bounds residual overlap, since genus-level masking leaves the correlated community structure intact.

### 2.8. Cohort Description, Class Handling, and Augmentation

Table 2 describes both cohorts as medians with interquartile ranges or counts with percentages. Standardized mean differences replace significance tests. Fibrinogen and C-reactive protein are descriptive only; referral cytology, histological grade, smoking, parity, intrauterine device use, lifetime partner number, and menopausal status enter as plausible confounders of the imaging branch or of vaginal composition. Ethnicity was not recorded. Imbalance was handled by stratified folds and focal loss without class weighting and calibration by temperature scaling.

Most standardized mean differences fall below 0.10; three exceed it, namely D-dimer (SMD 0.11), the derived ratio (0.13), and the proportion with CIN2 or worse (0.14), each higher at Center C. The external cohort therefore carries a modestly more advanced lesion spectrum, 26.4% with CIN2 or worse against 22.9% in development, together with slightly higher coagulation-immune values, which is consistent with a referral threshold that differs between institutions. All three imbalances are small in absolute terms, but the external estimates should be read as transfer to a case mix related to, rather than identical with, the one in which the model was trained. Training-fold images were augmented by random horizontal flipping, rotation within plus or minus 15 degrees, brightness and contrast jitter, and random cropping with resizing that preserves the transformation zone, with none applied to validation or external images. Mixup between paired same-class samples was applied to the tabular branch, and because mixup affects calibration, a variant trained without it appears in the component ablation.

Table 2 compares the two cohorts and therefore says nothing about how the coagulation-immune measures behave across the outcome categories themselves. Because the D-dimer-to-lymphocyte ratio is central to the study, its distribution and that of its two components are given by category in Table 3, pooling the development and external cohorts so that the co-infection group contains 102 rather than 26 participants. The contrast of interest, co-infection against no co-infection within HPV-positive women, is summarized beneath the table as a median shift and a rank-based effect size.

Contrast of primary interest, co-infection against no co-infection within HPV-positive participants (*n* = 336): Hodges-Lehmann median shift in the ratio 0.05 (95% CI 0.02 to 0.09); Cliff’s delta 0.22 (95% CI 0.10 to 0.34); Mann-Whitney *p* = 0.001. All comparisons are descriptive and uncorrected.

The ratio is shifted upward in the co-infection category, but the distributions overlap substantially, and the effect size is small to moderate, with Cliff’s delta of 0.22 corresponding to roughly a 61% probability that a randomly chosen participant with co-infection has a higher ratio than one without. In a logistic model restricted to HPV-positive participants and adjusted for age, histological grade, smoking, and HPV16 or HPV18 status, each doubling of the ratio was associated with an odds ratio for co-infection of 1.31 (95% CI 1.02 to 1.69). With 102 events and four covariates, this is at the limit of what the data support; it was fitted after evaluation was complete and is reported as descriptive.

### 2.9. Proposed Architecture

TIM-Fusion (Tri-modal Integration Model) uses a ConvNeXt-Tiny image encoder (28.6 million parameters), ImageNet-1k initialized and domain-adapted by MoCo v3 pretraining on unlabeled development-cohort images [19], fine-tuned with only the final stage and classification neck unfrozen; no external colposcopy dataset was used, precluding overlap with the external cohort. A feature-tokenizer transformer [20] (three layers, eight heads, 192 dimensions) takes the DLR and age. The microbiome branch is a 256-128-40 perceptron whose input concatenates the masked centered log-ratio genus vector with a one-hot encoding of community state type, the latter supplied as a compact summary of the overall community configuration alongside the individual genus abundances. Age is carried in this branch as a covariate rather than as a variable of interest and is retained in every arm of the parameterization comparison in Section 3.4, so that the differences reported there concern the coagulation-immune inputs only. The three 256-dimensional embeddings are scaled by confidence weights from a shared two-layer scoring head, learned end-to-end; an entropy regularizer (0.05) and modality dropout (0.15) prevent modality collapse and permit missing-input inference. Fusion is a bottleneck cross-attention layer [21] (4 latent tokens, 2 layers, 4 heads, dimension 256) where all three embeddings attend jointly rather than pairwise, followed by a classifier with dropout 0.3 and softmax over three categories (Figure 2, middle panel).

### 2.10. Baseline and Comparator Models

All comparators shared preprocessing, folds, seeds, and training budget, with independently tuned hyperparameters. The tabular-only baseline applies gradient-boosted trees to the DLR, age, and transformed genus vector as a flat feature set; the image-only baseline adds a classification head to the image encoder. Three pairwise ablations use the proposed encoders and fusion, so the DLR-plus-microbiome model differs from the tabular-only baseline only in architecture. Feature-level concatenation and pairwise cross-attention complete the internal set.

Three published architectures were reimplemented: the hybrid early fusion of Aquilina and Papagiannakis [11], the transformer fusion of Madathil et al. [12], and the Swin-transformer ensemble of Himabindu et al. [14]. The first reproduced an AUC of 0.74 against 0.75 as published, and the third an accuracy of 98.9% against 99.44%; the second, lacking public source data, was verified against its specification alone. Each was evaluated in its original two-modality form and in a three-modality extension carrying the microbiome vector.

Colposcopic assessment used the Swede score, an ordinal 0 to 10 instrument recorded at the visit by eleven colposcopists (median 9 years’ experience) blinded to laboratory and microbiome results. It was the decision variable for each one-versus-rest curve, reversed for the HPV-negative category. Colposcopists are not trained to identify co-infection from appearance, so this baseline shows what current practice recovers incidentally.

### 2.11. Training Configuration

Models were trained in PyTorch 2.2 with mixed precision. The objective was focal loss plus the entropy regularizer, optimized with AdamW under cosine annealing and early stopping on validation macro-AUC. Hyperparameters were selected by nested search within training folds, isolating the external cohort from tuning; probabilities were recalibrated by temperature scaling fitted on validation folds and applied unchanged externally. Table 4 gives the settings. Training and validation loss and macro-AUC were logged at every epoch of every fit and are plotted in Supplementary Figure S1.

Five-fold cross-validation across five seeds gave 25 models per configuration; external predictions were their mean softmax, identically for every comparator. Twenty-seven configurations at 25 fits each gave 675 runs and roughly 740 GPU-hours on an NVIDIA A100 (40 GB), with Ubuntu 22.04 LTS, CUDA 12.1, Python 3.10, scikit-learn 1.4, QIIME2 2023.9, shap 0.45, and R 4.3.2.

### 2.12. Evaluation Metrics

Performance was evaluated by accuracy, sensitivity, specificity, precision, F1 score, and AUC, computed per class one-versus-rest and macro-averaged without weighting. Calibration was assessed per class by intercept and slope with loess-smoothed curves [22]; the Hosmer-Lemeshow test was avoided as grouping-dependent and underpowered here. The multiclass Brier score uses the class-averaged convention:

$$BS=\left(1\right)/\left(N\right)\sum {\left(i=1\right)}^{\left(N\right)}\left(1\right)/\left(K\right)\sum {\left(k=1\right)}^{\left(K\right)}{\left({\hat{p}}_{\left(ik\right)}-y_{\left(ik\right)}\right)}^{2}$$

where K = 3, against the no-information score from predicted prevalences.

Net benefit [23] was computed over thresholds of 0.05 to 0.50 for a hypothetical decision to intensify follow-up, the threshold encoding the exchange rate between a missed co-infection and an unnecessary intensification. Input acquisition costs are excluded; references are the trivial strategies. Intervals derive from 2000 bootstrap resamples, bias-corrected and accelerated.

### 2.13. Interpretability

t-SNE on external fused embeddings is illustrative only, since structure depends on perplexity and initialization and inter-cluster distances are not quantitative; separability is quantified by silhouette coefficient and k-nearest-neighbor accuracy in the 256-dimensional space. GradientSHAP [24] (background 200, 500 samples per attribution) was applied at two levels, both on development-cohort participants only, keeping feature selection independent of the external cohort. At the modality level, attributions were taken with respect to each of the three 256-dimensional embeddings before fusion and summed within each embedding, giving one value per modality per participant; these support the comparison of modalities against one another. At the input level, attributions were taken with respect to the model inputs themselves, that is, the DLR, age, the centered log-ratio genus vector, and the one-hot community state type, with the target set to the logit of the participant’s own category. The two attributions are computed independently, and embedding-level values are not projected back onto input features: the encoders are not invertible, and any such mapping would be arbitrary. Spatial saliency for the image branch was computed by Grad-CAM [25], since ConvNeXt-Tiny is fully convolutional and contains no attention mechanism from which weights could be read directly. Gradients were taken with respect to the logit of the participant’s own category and pooled over the output feature map of the final ConvNeXt stage, the 768-channel 7-by-7 tensor immediately preceding the classification neck, which is the deepest layer retaining spatial correspondence. Channel-wise gradient means weighted that feature map; the weighted sum was passed through a rectified linear unit, and the result was normalized to the unit interval by per-image minimum and maximum, then upsampled bilinearly to the 224 by 224 input resolution. Iso-saliency contours are drawn at 0.35, 0.55, and 0.80 of the per-image maximum.. Each participant has 25 maps, one per fit; these were normalized individually and then averaged, so that no single fit dominates through a difference in scale. Maps were rated on a prespecified 4-point scale by three uninvolved gynecologic oncologists blinded to category and output; agreement was assessed by Fleiss’ kappa; being alterable without changing predictions, they are descriptive only.

### 2.14. Statistical Analysis

Per-class AUC differences used DeLong’s test [26]; because it does not extend to macro-averaged multiclass AUC, macro differences used paired bootstrap over 2000 resamples, the *p*-value and bias-corrected accelerated interval from one distribution. The prespecified family was all thirteen configurations under Holm control; colposcopist impression is reported for context but excluded, and per-class DeLong tests are nominal detail. One further analysis was added after peer review and is reported as a highlighted secondary result: a binary comparison restricted to HPV-positive participants, co-infection versus no co-infection, in 84 external participants. It lies outside the prespecified family, so its intervals and *p*-values are nominal and carry no multiplicity correction, and it is reported in Section Secondary Analysis: Co-Infection Within the HPV-Positive Stratum. Distributions of the coagulation-immune measures across outcome categories are described in Table 3, with the Hodges-Lehmann shift and Cliff’s delta as effect sizes and bootstrap intervals from 2000 resamples, and with a logistic model restricted to HPV-positive participants and adjusted for age, grade, smoking, and HPV16 or HPV18 status. All of these were specified after evaluation and are descriptive.

### 2.15. Subgroup and Confounder Analysis

Macro-AUC was computed within age, community state type, and histological grade strata and compared between Centers A and B; with 34 to 79 participants per subgroup, the analysis is underpowered. A multinomial model on the pooled 560, giving 102 rather than 26 events, tests whether output remains associated with co-infection after adjustment for grade, referral cytology, HPV16 or HPV18, smoking, and intrauterine device use; run only after evaluation was complete, its coefficient is read qualitatively.

### 2.16. Reporting Standards

Reporting follows TRIPOD+AI [27], as a diagnostic classification model, with a checklist as Supplementary Material. Inference time and trainable parameters are reported for the single model and ensemble. The study was not prospectively registered, though the outcome definition, partitioning, comparison family, and correction procedure were fixed before training.

## 3. Results

### 3.1. Participant Flow and Cohort Characteristics

Of 843 women assessed, 283 were excluded (Figure 1), most often for an incomplete same-visit triad (96); the remaining 560 formed the development cohort of 420 and the external test cohort of 140. Referral indications were abnormal cytology (61.4%), high-risk HPV with normal cytology (28.1%), and clinical suspicion or surveillance (10.5%). Median D-dimer lay within the reference range in both, and reviewer agreement on image admissibility gave Cohen’s kappa of 0.86.

### 3.2. Performance Across Validation and External Test Sets

Table 5 gives validation performance over five folds and five seeds with external performance, Wilson intervals for proportions and logit-scale for AUC; every configuration was trained 25 times and ensembled identically. Standard deviations understate uncertainty, as folds are not independent. Training accuracy exceeded validation by 0.02 to 0.05, and Supplementary Figure S1 plots training and validation loss on the left axis and training and validation macro-AUC on the right against epoch, one panel for each of the fourteen configurations of Table 5. Each curve is the mean over the 25 fits of that configuration, five folds by five seeds, taken at each epoch index over the fits still training at that point, with no interpolation and no padding to a common length. Because early stopping monitored validation macro-AUC with a patience of 15 epochs, fits ended at different epochs, and the dotted line and star in each panel together mark the selected epoch, the one at which mean validation macro-AUC was highest and whose weights were carried forward. Transfer loss ranged from 0.00 to 0.03, largest in the tabular-only baseline and the deepest comparator (validation 0.92), which, with thirteen configurations and one site, is compatible with sampling variation.

### 3.3. Modality Ablation

Table 5’s pairwise rows give each modality’s contribution. Imaging dominates: without it, DLR plus microbiome falls to 0.78, near the tabular-only level, whereas dropping either non-imaging modality stays above the image-only baseline. The DLR-plus-microbiome model and the tabular-only baseline receive identical information and differ by 0.02, the contribution of learned encoding. Microbiome and coagulation-immune inputs contribute comparably alongside imaging (0.86 and 0.85), and the full model exceeds the best pairwise combination by 0.06. For the reimplemented architectures, all three modalities changed macro-AUC by 0.01, 0.00, and −0.01; permuting the vector shifted probabilities by 0.04, so it is absorbed rather than exploited.

### 3.4. Parameterization of the Coagulation-Immune Branch

Three parameterizations of the coagulation-immune branch were compared. Age was present in the tabular branch in all three, and only the coagulation-immune inputs differed between them, so the differences below are not confounded by the presence or absence of age. All three lie outside the prespecified family, so intervals are nominal. The ratio alone gave 0.92 (0.86 to 0.96); D-dimer and lymphocyte count entered separately gave 0.89 (0.82 to 0.93), a difference of 0.03 (95% CI 0.01 to 0.06); and all three together, introducing collinearity, gave 0.90 (0.85 to 0.96), with the D-dimer contribution reversing sign in nine of 25 models. Being a deterministic function of its components, the ratio cannot carry information they lack; the difference is an estimation effect and a finite-sample observation, not evidence for the ratio as a construct. Stated as a conclusion rather than a caveat: these data do not establish that the ratio form is necessary. What they show is that supplying the quotient pre-formed spared the tabular branch from having to recover the same relationship from 420 development participants, and that the advantage of doing so was 0.03 macro-AUC with a lower bound of 0.01. In a larger development sample, the difference would be expected to shrink toward zero, and no result reported here would change if D-dimer and lymphocyte count were entered separately, since that configuration reaches 0.89 (0.82 to 0.93) and preserves the modality ordering in Table 5.

### 3.5. Architectural Component Ablation and Missing-Modality Behavior

Table 6 attributes the gain to individual components, each altered in turn, with its lower panel reporting inference with one modality withheld. Four differences exclude zero: replacing the bottleneck with concatenation costs 0.05, removing the bottleneck and weighting together 0.08, withholding the microbiome 0.06, and withholding the image 0.13. The 0.08 matches the sum of the two effects and the 0.84 of concatenation fusion in Table 5, consistent with additivity. Remaining components account for 0.01 to 0.03, with intervals spanning zero. One clarification is needed about what the withholding rows measure. Modality dropout operates at the level of the branch, so the row labelled inference with DLR withheld masks the entire tabular input, age included, and its 0.04 is therefore the contribution of that branch rather than of the ratio alone. Two further ablations separate the two. Retaining age and masking only the ratio gives 0.89 (0.82 to 0.94), a difference of 0.03 (95% CI −0.02 to 0.08) from the full model, while retaining the ratio and masking only age gives 0.92 (0.86 to 0.96), a difference of 0.00 (95% CI −0.04 to 0.04). Almost all of the branch effect is therefore attributable to the ratio, but neither interval excludes zero, and the attribution is not established. Both rows lie outside the prespecified family, and their intervals are nominal. Without the DLR, the full model reaches 0.88 against 0.86 for the purpose-trained image-plus-microbiome model on the same information, a difference of 0.02 (95% CI −0.03 to 0.07, *p* = 0.41) that is not established.

The row labelled inference with DLR withheld masks the whole tabular branch, age included, since modality dropout operates at branch level; the two final rows separate the ratio from age by masking one input at a time.

### 3.6. Per-Class Performance

Table 7 reports one-versus-rest class performance on the external cohort, each rate given with its TP, FP, and FN counts from the matrix in Supplementary Table S1; rows sum to 126 correct of 140, accuracy 0.90, with 14 false positives and 14 false negatives. Denominators of 56, 58, and 26 drive the interval widths; those for AUC are on the logit scale.Discrimination was strongest for the HPV-negative class and weakest for the co-infection class, the smallest and the one of interest. These are one-versus-rest figures, so the negative class of the co-infection row contains both the 58 HPV-positive women without co-infection and the 56 HPV-negative women, and the latter are the participants the model separates most easily. The co-infection AUC of 0.87 therefore overstates what the model achieves on the contrast of clinical interest. Section Secondary Analysis: Co-Infection Within the HPV-Positive Stratum reports that contrast directly, restricted to the 84 HPV-positive participants.

All rates are one-versus-rest, so each negative class comprises the two remaining categories; for the co-infection row, this includes the 56 HPV-negative participants. The binary co-infection analysis within the HPV-positive stratum alone is given in Table 8.

#### Secondary Analysis: Co-Infection Within the HPV-Positive Stratum

The 84 HPV-positive participants of the external cohort, 26 with and 58 without co-infection, were rescored as a binary problem by renormalizing the two HPV-positive class probabilities over their sum, so that no model was retrained, and no participant was discarded. Table 8 reports the resulting metrics at the default threshold of 0.5. Discrimination is lower than the one-versus-rest figure of 0.87 reported in Table 7, which is expected, since that formulation places 56 HPV-negative women in the negative class, and those are the participants whom the model separates most easily. The precision-recall area is reported against a prevalence of 0.31 in this stratum, and the calibration intercept and slope come from the same temperature parameter fitted on the validation folds and applied unchanged. With 26 events, every interval here is wide, and this analysis was added after peer review, so its *p*-values and intervals are nominal and uncorrected.

Prevalence of co-infection in this stratum was 0.31. Probabilities were obtained by renormalizing the two HPV-positive class probabilities of the three-class model over their sum; no model was retrained, and no participant was excluded. Counts differ from the co-infection row of Table 7 only in the false positives, six of which included one HPV-negative participant who does not enter this stratum. Intervals are from 2000 bias-corrected and accelerated bootstrap resamples; the analysis was added after peer review, and its intervals and *p*-values are nominal and uncorrected.

### 3.7. Statistical Comparison Against Comparators

Table 9 reports the thirteen prespecified comparisons, with differences and intervals derived from the same paired bootstrap distribution as the accompanying macro *p*-values. Under Holm correction across the full family, every comparison retained significance, including those against the three-modality forms of the reimplemented architectures. The smallest difference, 0.03 macro-AUC against the two-modality Swin ensemble, carries a nominal *p*-value of 0.031 and is retained because it is the largest of the thirteen ordered *p*-values, at which rank the Holm threshold equals the unadjusted alpha; it is the weakest of the thirteen, with a lower bound of 0.002 and its magnitude close to what repeated training of the same architecture can produce. That last point can be read directly from Table 5. The validation macro-AUC of the two-modality Swin ensemble was 0.92 with a standard deviation of 0.06 across the twenty-five fits, so a difference of 0.03 is half the spread that repeated training of that architecture produces on the same data. This comparison is therefore reported for completeness and is not read as establishing superiority over that architecture. It is also the only one of the thirteen with this property: recomputing Holm control over the remaining twelve comparisons leaves every one of them significant, the largest remaining macro *p*-value being 0.018, so no conclusion drawn in this paper rests on it.

The comparison against the two-modality Swin ensemble carries the largest of the thirteen ordered *p* values and a difference within the retraining variability of that architecture; it is retained for completeness and is not interpreted as established (Section 3.7).

### 3.8. Confusion Matrices and Receiver Operating Characteristic Curves

**Figure 3:**
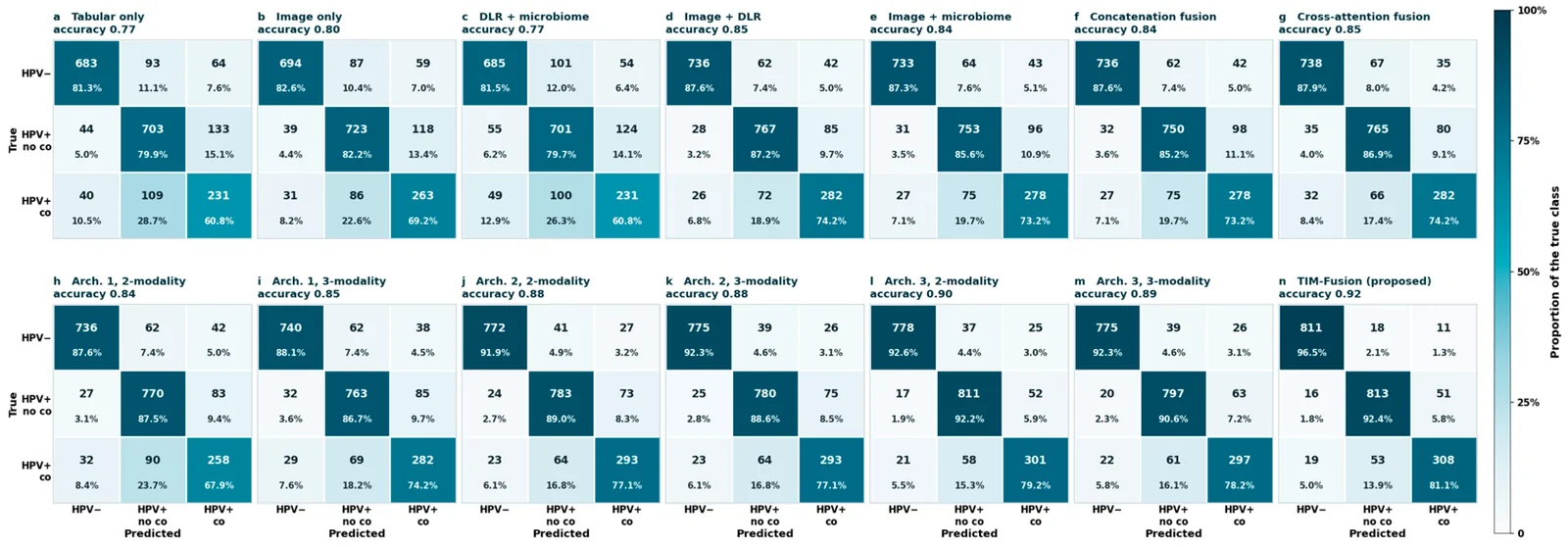
Confusion matrices by configuration, pooled cross-validation. Subplots (**a**–**n**).

**Figure 4:**
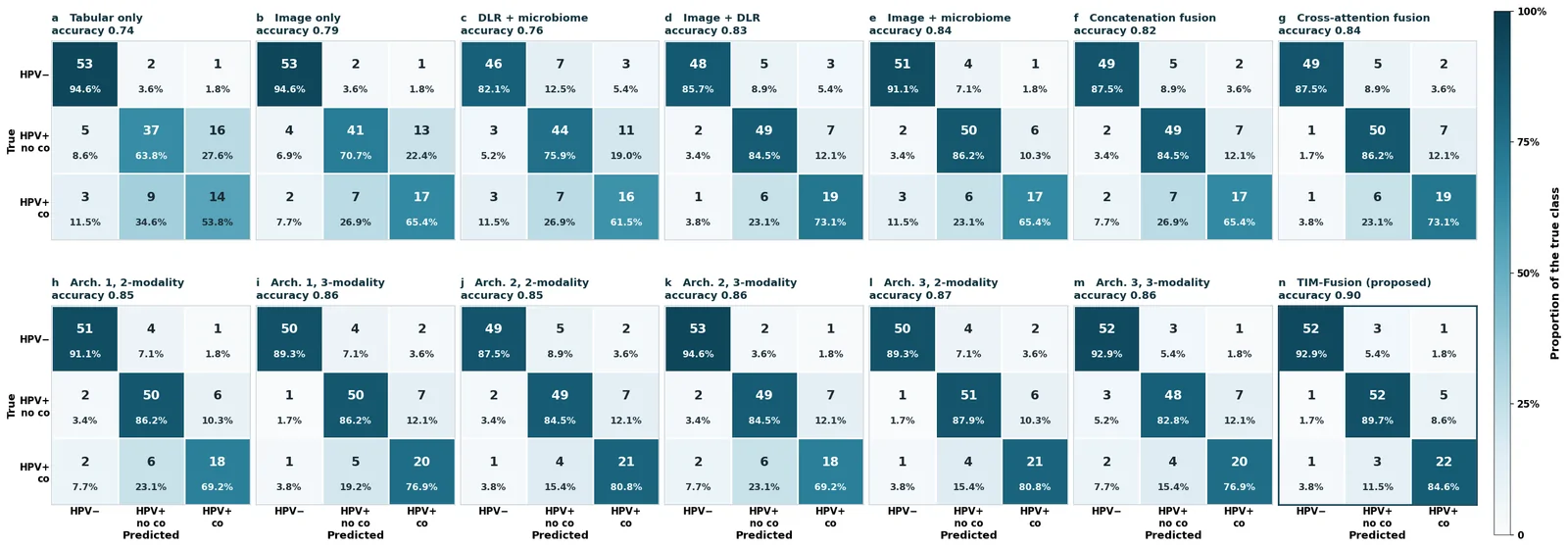
Confusion matrices by configuration, external test cohort. Subplots (**a**–**n**).

Figure 3 and Figure 4 give confusion matrices for every configuration as a two-by-seven grid of subplots labelled a to *n*, ordered as in Table 5 with the proposed model last and sharing a common color scale. Figure 3 covers pooled out-of-fold development predictions, in which each participant contributes five predictions, one per seed; Figure 4 covers the external cohort, and Supplementary Table S1 reproduces that matrix numerically. Across the panels, the comparators lose accuracy in the same cells rather than in different ones, with the additional errors falling mainly on the co-infection column.

**Figure 5:**
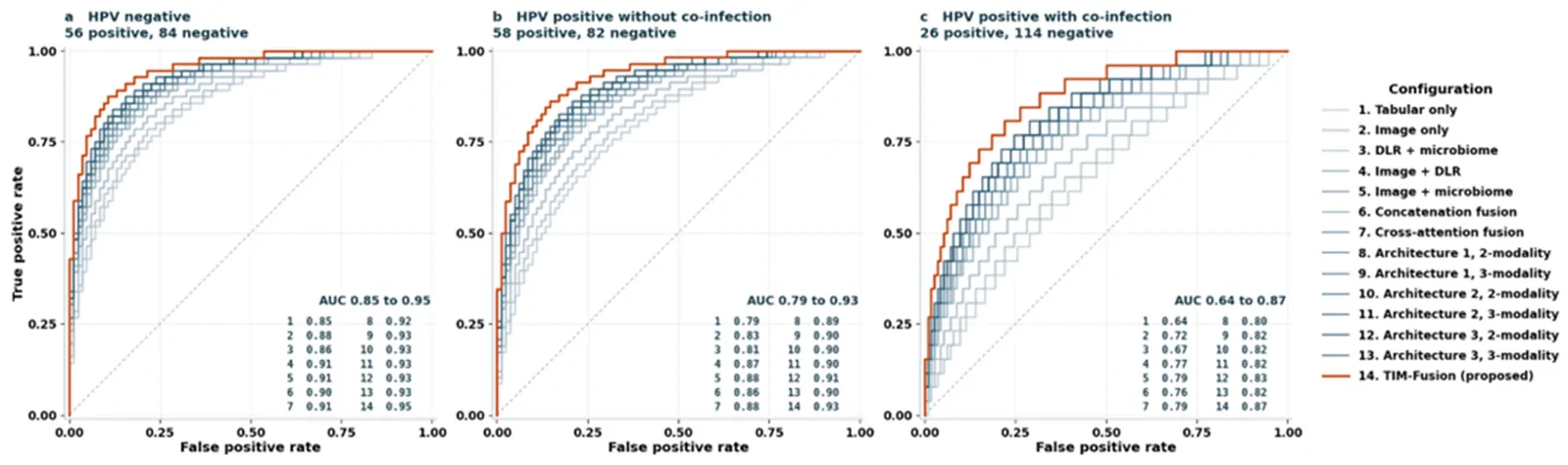
Receiver operating characteristic curves by class. Subplots (**a**–**c**).

Of the 14 external errors of the proposed model, eight fell between the two HPV-positive categories, five from HPV-positive without co-infection to the co-infection class and three in the reverse direction, while six involved the HPV-negative class. The margin between the two error types is narrow at these counts and should not be pressed. Figure 5 gives receiver operating characteristic curves as three subplots, one per category in a one-versus-rest formulation, each overlaying all fourteen configurations against the chance diagonal. The panels rest on denominators of 56, 58, and 26, so the co-infection panel is the least precisely estimated, and separation between configurations is widest there. The macro-averaged value reported throughout is the unweighted mean of the three areas.

### 3.9. Sensitivity to Taxon Masking, Organism, and Batch Correction

With Mycoplasma and Ureaplasma masked a priori, macro-AUC was 0.92 and co-infection-class AUC 0.87; additionally, masking the bacterial-vaginosis consortium gave 0.90 (0.84 to 0.94) and 0.83 (0.69 to 0.92). Shared susceptibility and proxy detection cannot be separated cross-sectionally, and genus-level masking bounds rather than resolves the concern; attributing the whole 0.04 reduction to proxy detection still leaves discrimination not resting primarily on surrogate taxa.

Per-organism counts appear in Supplementary Table S2; the 26 external cases comprised 11 *C. trachomatis*, 7 *T. vaginalis*, 6 *M. genitalium*, and 3 *N. gonorrhoeae*, 1 positive for two, leaving 10 with *C. trachomatis* alone. *T. vaginalis* leaves a community signature though absent from 16S: restricting the external co-infection class to the 19 participants without it reduced AUC from 0.87 to 0.82, a difference of 0.05 (95% CI −0.04 to 0.14, *p* = 0.28) that is not established. Conversely, separating the 10 with *C. trachomatis* alone from co-infection-free participants gave 0.79 (0.62 to 0.90) against 0.76 (0.58 to 0.88) for the image-only model. Omitting batch correction gave a macro-AUC of 0.91 (0.85 to 0.95), and Table 10 reports the full external performance in that configuration. The loss relative to the harmonized analysis is concentrated where it would be expected, in the co-infection class, whose AUC falls from 0.87 to 0.84 and whose sensitivity falls from 0.85 to 0.81, while the HPV-negative class is essentially unaffected. Within the HPV-positive stratum, the binary co-infection AUC falls from 0.86 to 0.83. Calibration also degrades, with the slope moving from 0.93 to 0.87, consistent with a shift in the input distribution that the model was not adjusted for. Since MMUPHin requires a batch of samples rather than a single patient, these uncorrected values are the deployment-relevant estimates, and the harmonized values in Table 5 and Table 7 should be read as the upper bound obtainable when a full external batch is available for harmonization. Complete-case analysis, excluding 16 participants, gave 0.92 (0.86 to 0.96).

Corrected values reproduce Table 5, Table 7 and Table 8. Uncorrected values apply the same 25 trained models to centered log-ratio abundances computed without MMUPHin harmonization; no model was retrained.

### 3.10. Calibration and Clinical Utility

**Figure 6:**
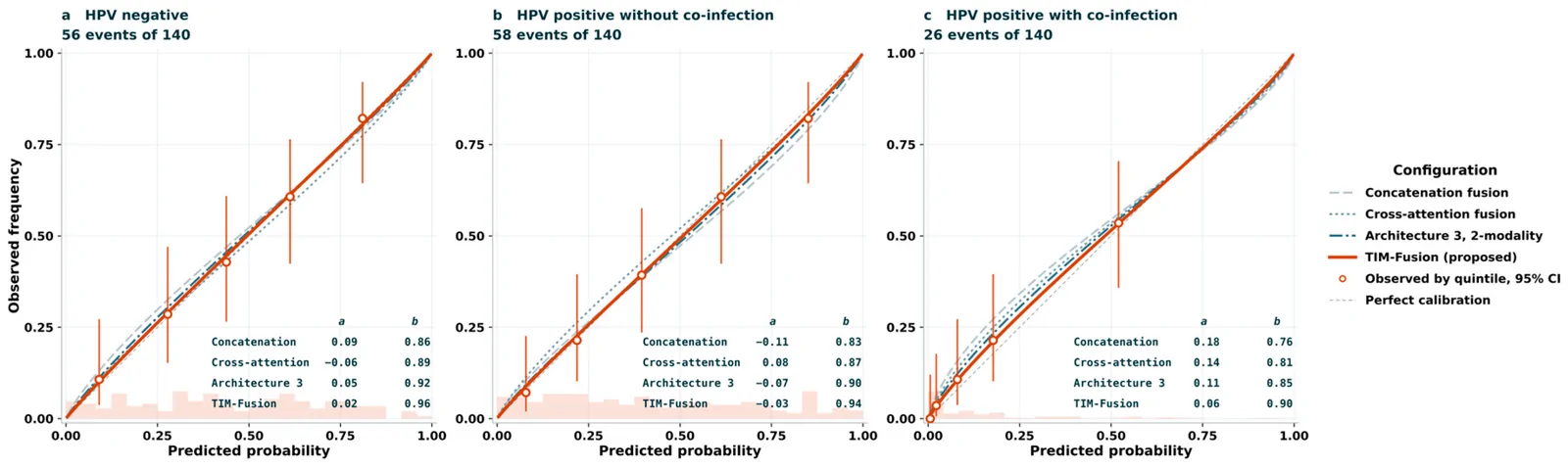
Calibration curves by class, external test cohort. Subplots (**a**–**c**).

Calibration after temperature scaling appears in Figure 6 and Table 11. Figure 6 gives one subplot per category, each showing the proposed model, both simple fusion strategies, and the strongest reimplemented architecture. The temperature parameter, fitted on validation folds and applied unchanged externally, yields these transfer estimates. Intercepts were near zero and slopes near unity in all three categories, with the widest interval and lowest slope in the co-infection category (26 events). The multiclass Brier score was 0.07 against a no-information score of 0.21.

**Figure 7:**
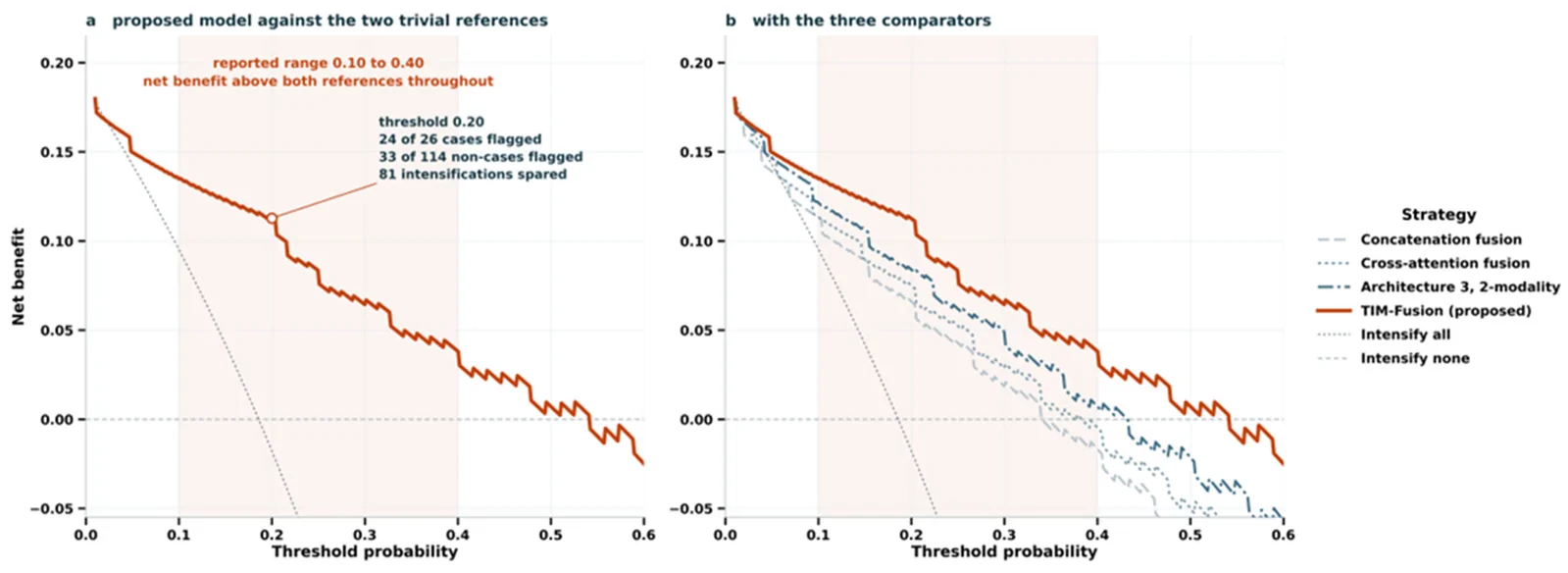
Decision curves for the co-infection category. Subplots (**a**,**b**).

Decision curve analysis appears in Figure 7 as two subplots, the first showing the proposed model against the intensify-all and intensify-none references and the second adding the same three comparators shown in Figure 6. The proposed model showed net benefit exceeding both trivial references across threshold probabilities from approximately 0.10 to 0.40, and the comparators followed the same shape at slightly lower net benefit.

At a threshold of 0.20, the co-infection rule reached sensitivity 0.92 and specificity 0.71, identifying 24 of 26 cases and intensifying 33 of 114 non-cases, sparing 58 intensifications per 100 women for two missed cases. The reference here is not intensify-all but the four-organism panel, which would dominate at every threshold, so the curve is informative only where that panel is unavailable.

### 3.11. Reduced-Input Configuration

Because the microbiome input is slowest and costliest, a reduced configuration using the ten highest-attribution taxa together with community state type was retrained in silico, with selection from development-cohort attribution only. Macro-AUC was 0.90 (0.84 to 0.94) and co-infection-class AUC 0.85 (0.72 to 0.93) against 0.92 and 0.87, a difference of 0.02 (95% CI −0.01 to 0.05), so ten taxa and community state type carry most of the branch’s contribution. Without quantitative PCR, this is a reduced-input analysis, not validation of an assay.

### 3.12. Embedding Structure, Attribution, and Modality Weighting

**Figure 8:**
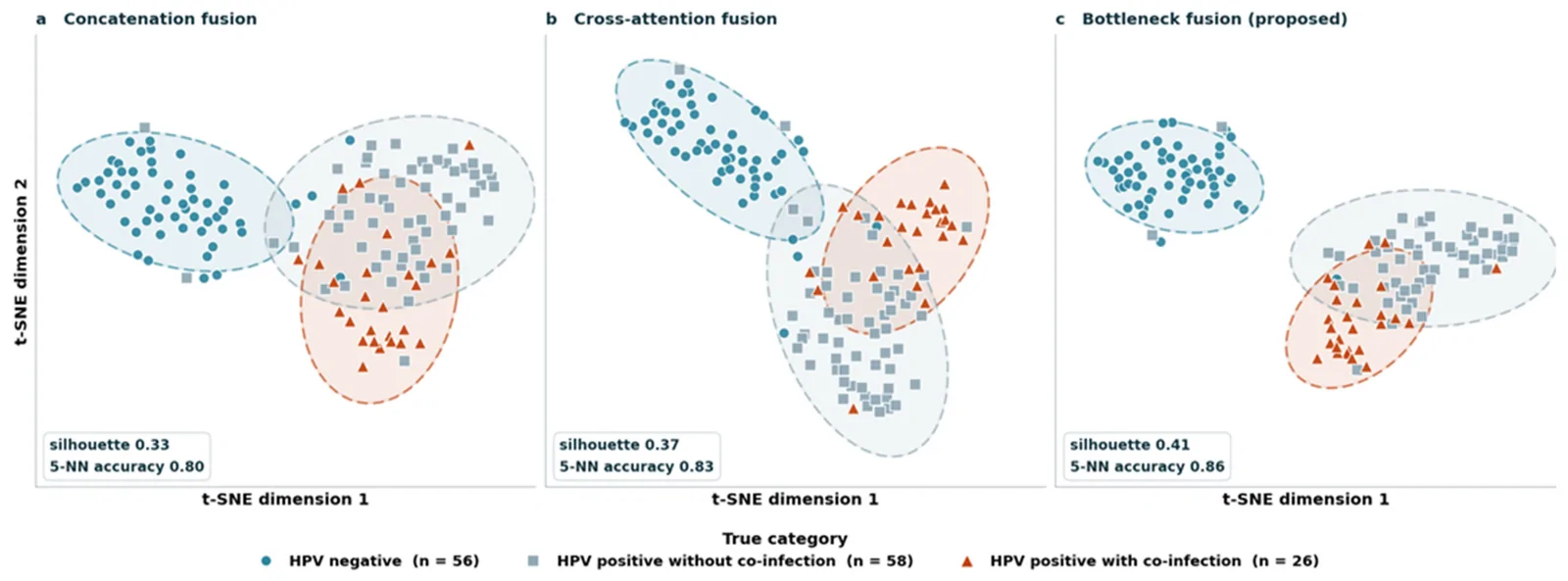
t-SNE projections of fused representations by fusion strategy. Subplots (**a**–**c**).

Figure 8 gives t-SNE projections for concatenation fusion, pairwise cross-attention, and bottleneck fusion on the same external participants. Overlap between the two HPV-positive classes narrows across the panels: silhouette rises from 0.33 to 0.37 to 0.41 (0.33 to 0.49) and k-nearest-neighbor accuracy from 0.80 to 0.83 to 0.86 (0.80 to 0.91).

**Figure 9:**
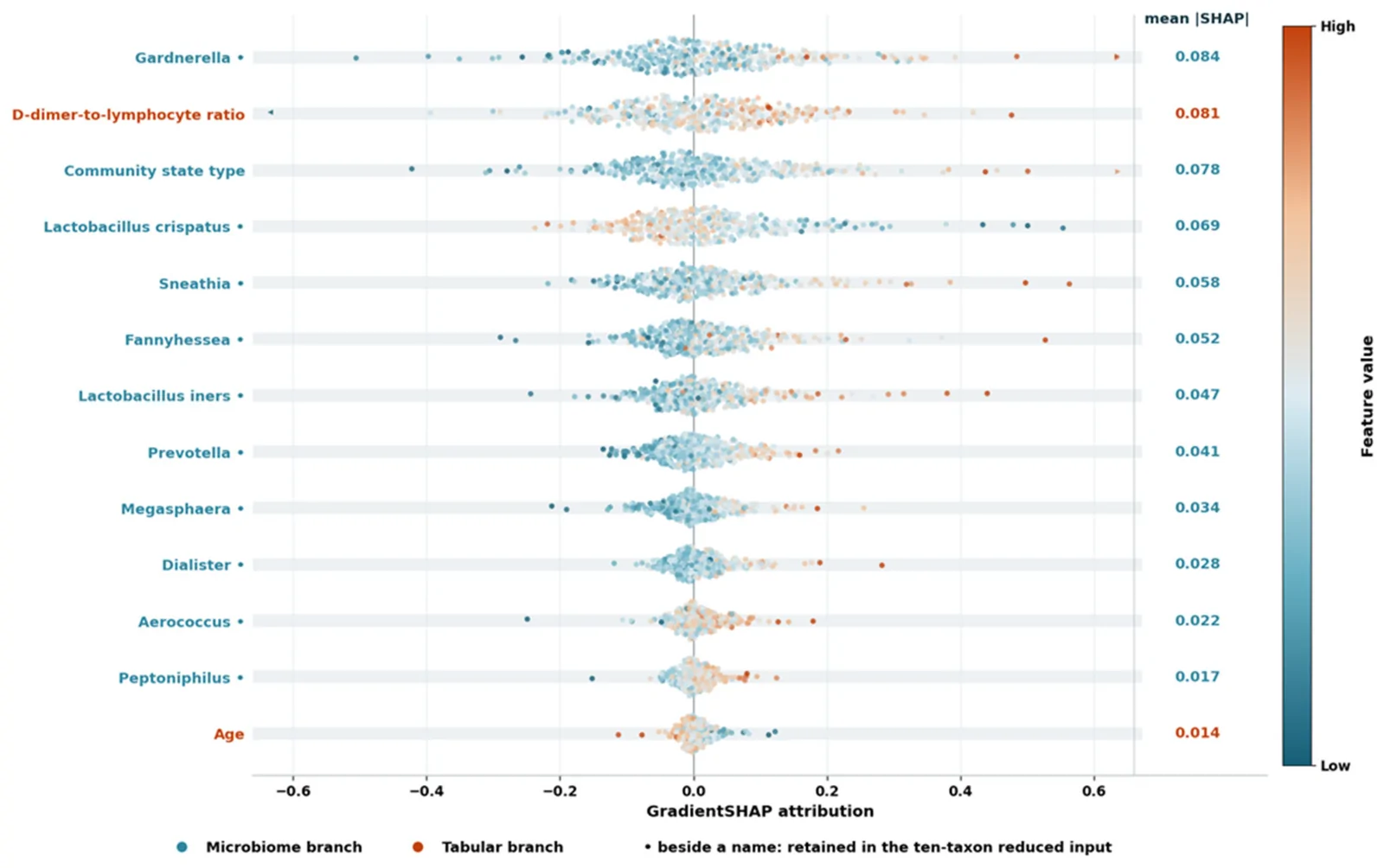
GradientSHAP attribution summary. Attributions are computed with respect to the model inputs, namely the DLR, age, the centered log-ratio genus abundances, and the one hot community state type, with the target set to the logit of each participant’s own category, on development-cohort participants only. Modality-level attributions, computed separately with respect to the three pre-fusion embeddings and reported in Section 3.12, are not derived from the values shown here.

Input-level GradientSHAP attribution, in Figure 9, placed the DLR, the larger of the two tabular contributions, on a par with the leading microbiome features: its mean absolute attribution of 0.081 sits between Gardnerella at 0.084 and community state type at 0.078, with Lactobacillus crispatus at 0.069 next. Age contributes the least of the fourteen inputs shown, at 0.014. Attribution of this kind indicates what the model relies on, not causal contribution. Learned modality confidence weights averaged 0.46 for imaging, 0.30 for microbiome, and 0.24 for coagulation-immune input, shifting toward the microbiome branch for the co-infection class; the fixed-weight ablation in Table 6 puts the value of that adaptivity at 0.03 macro-AUC, with an interval spanning zero.

### 3.13. Saliency Maps, Subgroups, and Lesion Grade

**Figure 10:**
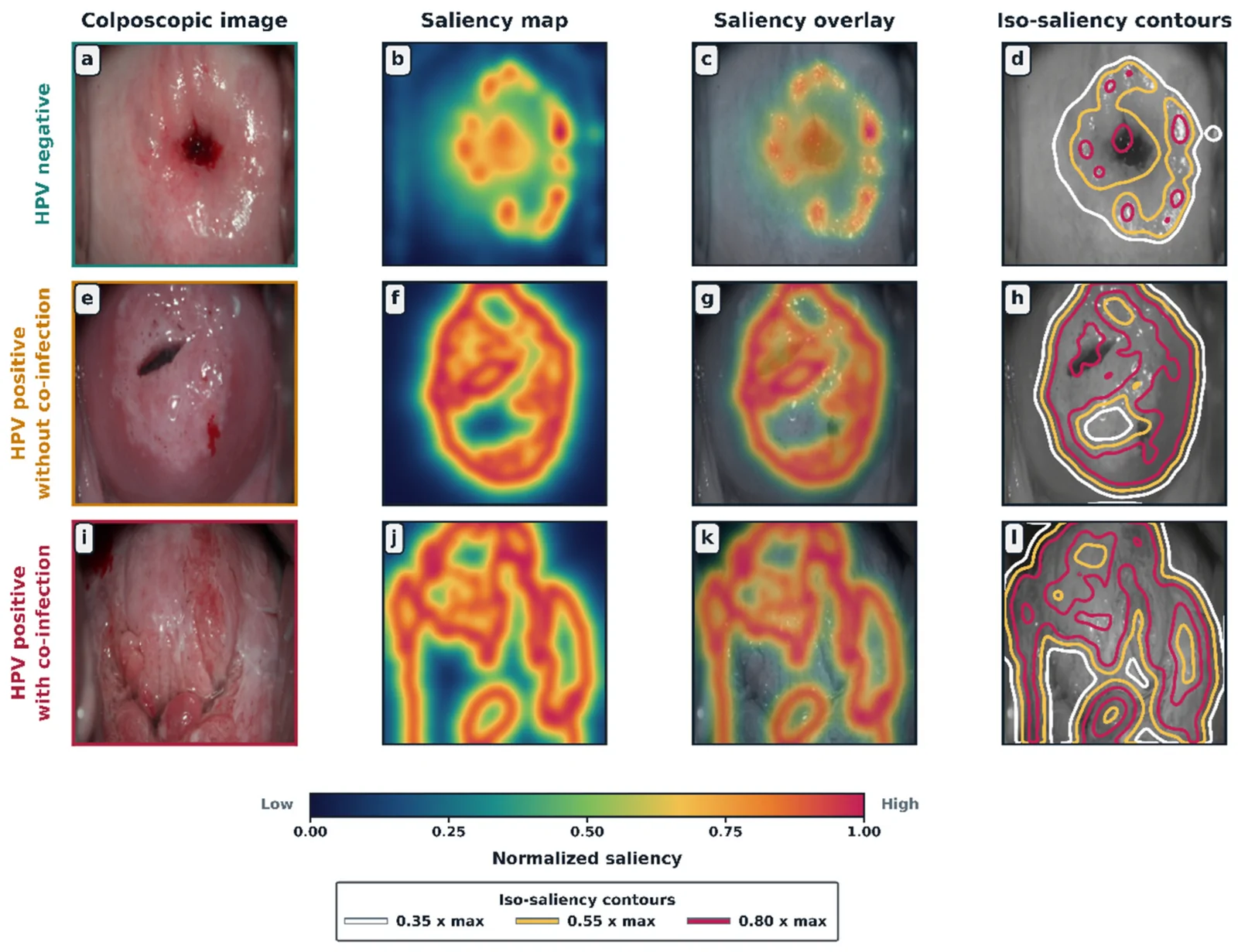
Grad-CAM saliency on colposcopic images. One representative participant per category: HPV-negative (**a**–**d**), HPV-positive without co-infection (**e**–**h**), and HPV-positive with co-infection (**i**–**l**). Columns show the colposcopic image, the saliency map, the map overlaid on the image, and iso-saliency contours at 0.35, 0.55, and 0.80 of the per-image maximum. Maps are computed at the output of the final ConvNeXt stage with respect to the logit of each participant’s own category, rectified, normalized per image to the unit interval, upsampled bilinearly to 224 by 224, and averaged over the 25 fits after individual normalization. Because normalization is per image, color indicates relative weight within an image and is not comparable in absolute terms between images.

Figure 10 overlays Grad-CAM saliency for the image encoder, with weight falling predominantly on acetowhite epithelium and abnormal vessels within the transformation zone. Of 140 maps rated by three blinded oncologists on a prespecified 4-point scale, 78.6% were plausible or highly plausible (Fleiss’ kappa 0.61). Saliency of this kind can be altered without changing predictions, so this does not establish the decision basis and is reported as a plausibility check rather than as evidence. Table 12 reports subgroup performance. Point estimates were similar across age and community state type, and discrimination held within each histological grade stratum, including women with no intraepithelial neoplasia, where severity cannot be what is encoded. The genotype strata are HPV-positive only, so those rows give binary co-infection AUC, 0.88 for HPV16 or HPV18 against 0.86 for other high-risk genotypes.

In the multinomial model on the pooled 560, output remained associated with co-infection after adjustment for grade, cytology, HPV16 or HPV18, smoking, and intrauterine device use (adjusted OR 1.68 per 0.1 increase in predicted probability, 95% CI 1.33 to 2.12). Out-of-fold macro-AUC was 0.94 (0.90 to 0.98) at Center A and 0.92 (0.86 to 0.96) at Center B. With 34 to 79 participants and 8 to 15 events per subgroup, smaller disparities cannot be excluded.

### 3.14. Computational Cost

A single trained model contained 31.4 million parameters, approximately 4.6 million updated during fine-tuning, requiring 42 ms per patient on the A100 configuration and 310 ms on a single CPU core. The deployed ensemble requires 25 forward passes, giving 1.1 s per patient on GPU and 7.8 s on CPU, with a stored size of approximately 143 million parameters rather than 25 times 31.4 million, because the frozen backbone is shared. The best single model achieved 0.91 (0.85 to 0.95) and the mean across the 25 was 0.90 (0.84 to 0.94), so ensembling contributes 0.01 to 0.02 at 25 times the inference cost; because every comparator was ensembled identically, this affects the absolute level of all entries in Table 5 rather than the differences. The ensemble is therefore a research artefact rather than a deployment configuration: the single-model estimate is the one a future study should carry, at 42 ms per patient on GPU and 310 ms on one CPU core.

## 4. Discussion

### 4.1. Principal Findings

On external validation, the fused model outperformed unimodal baselines, all pairwise combinations, both simple fusion strategies, and the three reimplemented architectures in original and three-modality form, all thirteen prespecified comparisons surviving Holm correction, with the caveat that the weakest of them, against the two-modality Swin ensemble, falls within the retraining variability of that architecture and is not counted as established. Two levels of evidence should be kept apart throughout. The macro-averaged figures describe separation of three categories, and most of that separation is the distinction between HPV-negative and HPV-positive women, which is settled by genotyping before the model is consulted. The evidence specific to co-infection is narrower: 26 external events, the lowest class-specific AUC of the three at 0.87 one-versus-rest, and 0.86 in the binary analysis restricted to HPV-positive participants. Every statement below about co-infection rests on that smaller body of evidence, and the difference between the two levels is not a discrepancy but a difference in what is being asked. Imaging is the dominant contributor, yet neither non-imaging modality is redundant given the other. Supplying the ratio pre-formed is worth 0.03, necessarily an estimation effect. Ablation attributes the architectural gain to the bottleneck (0.05, interval excluding zero); learned modality weighting adds 0.03 with an interval spanning zero. Read within the HPV-positive stratum alone, where the negative class no longer contains HPV-negative women, discrimination of co-infection is more modest than the three-class macro figure, and Section Secondary Analysis: Co-Infection Within the HPV-Positive Stratum reports that contrast directly.

### 4.2. What the Model Appears to Have Learned

Imaging carries most of the HPV-status signal, while the microbiome branch carries much of the co-infection signal. The contribution is uneven across organisms, largest for T. vaginalis and near absent for *C. trachomatis*, where discrimination falls to the image-only level; non-redundancy is a statement about the co-infection class, not any individual pathogen. The marginal distributions in Table 3 make the same point from the other direction: the ratio separates the two HPV-positive groups only weakly on its own, so what the model recovers from it is a contribution conditional on imaging and microbial context rather than a standalone association.

Residual confusion falls between the two HPV-positive categories in 8 of 14 external errors, consistent with co-infection modifying the mucosal environment without a distinct colposcopic signature. Masking indicates discrimination does not rest on surrogate taxa, and preserved discrimination among women with no intraepithelial neoplasia indicates the imaging branch is not encoding lesion severity. Within the HPV-positive stratum alone, the binary co-infection AUC is 0.86 (Table 8), so most of the macro-averaged discrimination reported throughout comes from separating HPV-negative women rather than from the co-infection contrast.

### 4.3. Relation to Blood-Biomarker Studies

Recent work has examined systemic inflammatory and coagulation ratios in cervical disease almost exclusively as single unimodal indices, as summarized in Table 13. Zhuang et al. [5] confirmed across 38 cohorts and 10,246 patients that elevated pretreatment neutrophil-to-lymphocyte ratio was associated with reduced overall survival; Chen JLY et al. [28] and Yu et al. [29] reported comparable associations with survival and treatment response, and Tietie et al. [30] and Chen Q et al. [6] reported clinical utility for plasma D-dimer. Each concerns patients with established carcinoma and treats the marker as a standalone variable read against a threshold, whereas the present design measures its contribution once imaging and microbiome information are already available.

Two estimates of that contribution diverge: withholding the DLR at inference costs 0.04 with an interval spanning zero, whereas training a model without it and comparing against the full model gives 0.06 (0.02 to 0.11). The difference is expected rather than contradictory, since the first perturbs a network that learned to use the input and can partly compensate through the remaining branches, while the second compares two separately optimized models. The second answers the question of incremental value, and on that basis a coagulation-immune index appears to retain some value at this earlier point in the disease spectrum, where D-dimer lies predominantly within the reference range.

### 4.4. Relation to Vaginal Microbiome Studies

Musa et al. [31] found non-Lactobacillus-dominant, high-diversity community types associated with high-grade precancer and invasive cancer, with high-risk HPV independently associated at an adjusted OR of 2.72; Bautista et al. [7] synthesized evidence that transition toward Gardnerella-enriched and Sneathia-enriched states modulates HPV persistence; and Ye and Qi [8] concluded that Lactobacillus depletion increases susceptibility to persistent infection.

The present study operationalizes those proposals by encoding taxa abundance as a model input rather than a descriptive covariate and shows where the contribution comes from: the community signature is informative for organisms that restructure the community and not for an intracellular pathogen that does not, which bounds how far microbiome profiling can substitute for targeted detection. It also confronts a difficulty descriptive work does not encounter, since when the microbiome is both an input and part of what defines the outcome, discrimination and overlap must be separated by design rather than post hoc.

### 4.5. Relation to Imaging-Based and Multimodal Studies

Aquilina and Papagiannakis [11] trained a classifier on 4946 histology-matched colposcopic images, reporting an average AUC of 0.75, falling to 0.70 across camera types and rising to 0.81 once image-based predictions were combined with age and referral data. Wang et al. [32] reported an accuracy of 96.18% across a multi-center cohort; Pacal and Kılıçarslan [33] compared more than 60 architectures on a public cytology dataset and underscored the difficulty of generalizing across imaging sources; and Madathil et al. [12] and Himabindu et al. [14] reported AUC of 0.999 and an accuracy of 99.44% on internal validation. The present results illustrate the contrast: the deepest reimplemented architecture achieved the highest validation performance of any comparator, 0.92, and lost 0.03 on transfer, the largest drop of any deep configuration.

Table 13 places the present work alongside representative studies from the three literatures it draws on. The rows differ in population, endpoint, data type, and reported metric, so values are not comparable across them and no ranking should be inferred; the purpose is to show where a tri-modal model sits relative to three bodies of work that have remained separate. Validation type is given because that column, rather than the metric column, explains most of the spread, with the highest reported figures coming almost entirely from internal validation on a single source. The blood-biomarker rows are drawn from populations with established carcinoma, whereas the present cohort is a colposcopy-referral population, a difference bearing directly on how far those findings transfer.

### 4.6. Interpretation and the Limits of Clinical Use

The results are best read as evidence about biology rather than as a proposal for practice. What they establish is that a systemic coagulation-immune index, local microbial ecology, and visible epithelial morphology carry partially non-redundant information about concurrent lower genital tract co-infection, and that the fusion architecture as a whole extracts more of that information than concatenation or pairwise attention, although the ablation does not resolve which of its components is responsible. That is a statement about how the three compartments relate, and it stands independently of whether any model built on them is deployable.

The deployment case does not hold, for a reason that should be stated directly. The outcome is the result of a four-organism nucleic acid amplification panel that is cheaper, faster, more available, and more accurate than the model that predicts it. Where that panel can be run, no model output should displace it, and the earlier suggestion that a graded probability might instead guide follow-up interval fails for the same reason: once the panel has been run, its result, not a probability, is the correct basis for that decision. The decision curve reported above is meaningful only in the narrow setting where the panel is unavailable while sequencing, colposcopy, and a coagulation panel are not, a combination that is uncommon and that this study did not sample. The disproportion between the computational effort and the practical yield is real and worth stating plainly. Training 675 models was the price of the scientific question rather than of the product: component-level ablation, thirteen comparisons under family-wise correction, and five-seed repetition are what allow the non-redundancy claim to be made with intervals attached, and none of that apparatus would be required to run the model itself. The finding that a four-organism panel resolves the same outcome more cheaply is the result, not a shortfall of it, and the appropriate return on this investment is a study design rather than a deployment.

Two observations retain practical value without depending on that setting. The reduced-input analysis shows that ten taxa together with community state type carry most of the microbiome branch’s contribution, which is a prerequisite for any future assay built on this signature rather than a demonstration of one. And the ablation identifies where the information sits, so that a prospective study of a genuinely prognostic outcome, such as persistence or clearance over 12 to 24 months, could be designed around imaging and microbiome sampling without carrying the coagulation panel unless its incremental contribution is replicated. The same argument applies twice over to HPV status. Its polymerase chain reaction result defines two of the three categories and is available in this pathway before the colposcopy that supplies the images, so the HPV-negative-versus-positive component of the reported discrimination answers a question that is already answered at the point of care. It is retained because it fixes the class structure and constrains what the imaging branch can be encoding, not because it has any standing as a prediction task. The reader should therefore treat the macro-averaged figures as a property of the analysis and the binary estimate in Table 8 as the one that corresponds to a clinical question, and even that question is answered more accurately by the four-organism panel.

### 4.7. Limitations

The present analysis is unusually complete for a study of this kind: three modalities acquired within a single visit, a fully independent external center, thirteen prespecified comparisons under family-wise correction, component-level ablation, missing-modality inference, and prespecified sensitivity analyses for input-outcome overlap. What remains open is mostly a matter of scope rather than execution. The external cohort comprises 140 participants from a single institution, 26 of whom carry the co-infection outcome, and two consequences follow. First, the class of primary interest is imprecisely estimated: its AUC of 0.87 carries a 95% interval of 0.75 to 0.94, an interval compatible with performance appreciably below the point estimate, and the same imprecision affects the calibration slope in that class. Under the external-validation criteria applied in Section 2.3 [16], the cohort meets neither the events nor the non-events requirement for a precise class-specific estimate. Second, a single external site fixes one combination of colposcopy unit, sequencing platform, and coagulation assay, so institutional and geographical transportability is tested only for the transfer from Centers A and B to Center C and cannot be assumed beyond it. Confirmatory claims are accordingly confined to macro-averaged discrimination. The macro-averaged figure and the binary estimate in Table 8 answer different questions, and the gap between them, 0.92 against 0.86, is a property of the class structure rather than a discrepancy. The population also entered through colposcopy referral, and the outcome is concurrent with the inputs rather than prognostic. That referral pathway matters for how the results should be read. Every participant had already been selected by abnormal cytology, a positive high-risk HPV result, or clinical suspicion, so the prevalence of both HPV-positivity and co-infection is far above what an unselected screening population would show. Discrimination is comparatively insensitive to that shift, but predictive values and net benefit are not, and the decision curve in Section 3.10 should not be transported to a screening setting. The three covariates with standardized mean differences above 0.10, D-dimer, the ratio, and the CIN2-or-worse proportion, all lie higher in the external cohort, so the transfer reported here was made to a slightly more advanced case mix rather than a matched one. Every microbiome-dependent estimate in this paper carries the same caveat, and Table 10 quantifies it: harmonization is worth 0.01 macro-AUC overall but 0.03 in the class that depends on it most. Each of these defines a well-specified next study. The most valuable is longitudinal: baseline tri-modal profiling with 12- to 24-month follow-up for HPV persistence or clearance, which converts the task to prognosis and separates shared susceptibility from concurrent overlap in a way cross-sectional data cannot. Beyond that, prospective multi-site collection powered to external-validation criteria, direct measurement of agreement between amplicon-derived and quantitative PCR abundance for the ten selected taxa from which community state type is derived, recording of ethnicity and menstrual cycle phase to support fairness assessment, and evaluation under Lugol iodine protocols would each extend these findings without unsettling their basis.

### 4.8. Future Directions

The natural successor study is longitudinal: baseline multimodal profiling with 12- to 24-month follow-up for HPV persistence or clearance, which would convert the task from diagnostic to prognostic, separate shared susceptibility from concurrent overlap in a way cross-sectional data cannot, and test whether the signature anticipates outcomes not already measurable at baseline. Beyond that, the priorities are prospective multi-site collection at a scale meeting external validation criteria and direct measurement of agreement between amplicon-derived and quantitative PCR abundance for the ten selected taxa from which community state type is derived, before any reduced-input configuration is deployed.

## 5. Conclusions

Fusing a coagulation-immune index, vaginal microbiome composition, and colposcopic imaging reached a macro-AUC of 0.92 (0.86 to 0.96) across three categories on an independent external cohort, ahead of every comparator after Holm correction. Discrimination of co-infection specifically was lower and less certain, 0.87 (0.75 to 0.94) one-versus-rest and 0.86 (0.76 to 0.93) within the HPV-positive stratum, on 26 events. Of the architectural components, only the bottleneck fusion layer has an effect whose interval excludes zero, at 0.05; confidence weighting, the entropy regularizer, modality dropout, the bottleneck token count, and mixup each account for 0.01 to 0.03 with intervals spanning zero. Supplying the D-dimer-to-lymphocyte ratio pre-formed is worth 0.03 macro-AUC, an estimation effect rather than evidence that the quotient carries information its parts do not.

Coagulation-immune status, local microbial ecology, and visible epithelial morphology therefore carry partially non-redundant information about HPV-associated co-infection, and this, rather than any clinical application, is what the study establishes. Confirmatory claims are confined to macro-averaged discrimination, which is the level at which the comparisons were prespecified and corrected. The co-infection class is where the clinical interest lies and where the evidence is thinnest: with 26 external events, performance in that class and the incremental value of the ratio form are consistent with the hypothesis but not demonstrated, and no reader should carry the macro figure across to that question. External validation rests on one center, so transportability across institutions and acquisition platforms is likewise untested. Restricting the microbiome branch to ten taxa and community state type preserves most of the signal in silico, identifying a route to a future assay without demonstrating one. Because the outcome is resolved directly by a cheaper and more accurate laboratory panel, the appropriate next step is a longitudinal design with an outcome that cannot be measured at baseline, not deployment of this model. All estimates are made in a colposcopy-referral population and do not extend to primary screening.

## Figures and Tables

**Table 1:** Inclusion and exclusion criteria.

Category	Criteria
Inclusion	Age 18 to 65 years
Inclusion	Colposcopy, blood panel, and vaginal swab obtained within the same visit window
Inclusion	Interpretable colposcopic image of the transformation zone
Inclusion	Documented HPV status confirmed by polymerase chain reaction testing
Inclusion	Complete and determinate co-infection panel result
Exclusion	Active non-genital systemic infection
Exclusion	Known coagulation disorder, or anticoagulant or antiplatelet therapy
Exclusion	Pregnancy or prior hysterectomy
Exclusion	Active malignancy at another anatomical site
Exclusion	Systemic antibiotic or intravaginal antimicrobial exposure within 30 days
Exclusion	Incomplete records or failed 16S sequencing quality control
Exclusion	Colposcopic image judged unreadable by independent reviewers
Exclusion	Persistently indeterminate co-infection panel result
Exclusion	HPV-negative with concurrent co-infection

**Table 2:** Cohort characteristics, development, and external test sets.

Characteristic	Centers A and B (Development, *n* = 420)	Center C (External Test, *n* = 140)	SMD
Age, years	34.1 (28.6 to 40.9)	34.8 (29.0 to 41.4)	0.06
Current smoker, *n* (%)	131 (31.2)	44 (31.4)	0.00
Parity of one or more, *n* (%)	271 (64.5)	88 (62.9)	0.03
Intrauterine device in situ, *n* (%)	61 (14.5)	23 (16.4)	0.05
Five or more lifetime partners, *n* (%)	149 (35.5)	54 (38.6)	0.06
Postmenopausal, *n* (%)	38 (9.0)	15 (10.7)	0.06
HPV-negative, *n* (%)	168 (40.0)	56 (40.0)	0.00
HPV-positive without co-infection, *n* (%)	176 (41.9)	58 (41.4)	0.01
HPV-positive with co-infection, *n* (%)	76 (18.1)	26 (18.6)	0.01
D-dimer, mg/L FEU	0.38 (0.27 to 0.54)	0.41 (0.28 to 0.59)	0.11
Absolute lymphocyte count, ×10^9^/L	1.92 (1.58 to 2.31)	1.86 (1.51 to 2.28)	0.09
D-dimer-to-lymphocyte ratio	0.20 (0.14 to 0.30)	0.22 (0.15 to 0.33)	0.13
Fibrinogen, g/L (available in 85.7%)	3.1 (2.7 to 3.6)	3.2 (2.7 to 3.8)	0.08
Platelet count, ×10^9^/L	254 (218 to 291)	249 (213 to 288)	0.07
C-reactive protein, mg/L (available in 81.4%)	1.8 (0.9 to 3.4)	2.0 (0.9 to 3.7)	0.06
HPV16 or HPV18 among HPV-positive, *n* (%)	98 (38.9)	34 (40.5)	0.03
Other high-risk genotype among HPV-positive, *n* (%)	154 (61.1)	50 (59.5)	0.03
Lactobacillus-dominant community state type, *n* (%)	245 (58.3)	79 (56.4)	0.04
Referral cytology: normal, *n* (%)	118 (28.1)	39 (27.9)	0.00
Referral cytology: low-grade, *n* (%)	178 (42.4)	60 (42.9)	0.01
Referral cytology: high-grade, *n* (%)	124 (29.5)	41 (29.3)	0.00
Histology: no CIN, *n* (%)	185 (44.0)	57 (40.7)	0.07
Histology: CIN1, *n* (%)	139 (33.1)	46 (32.9)	0.00
Histology: CIN2 or worse, *n* (%)	96 (22.9)	37 (26.4)	0.14

**Table 3:** Coagulation-immune measures by outcome category, pooled development, and external cohorts (*n* = 560). Values are median (IQR).

Measure	HPV-Negative (*n* = 224)	HPV-Positive Without Co-Infection (*n* = 234)	HPV-Positive with Co-Infection (*n* = 102)
D-dimer, mg/L FEU	0.36 (0.26 to 0.50)	0.38 (0.27 to 0.55)	0.45 (0.31 to 0.63)
Absolute lymphocyte count, ×10^9^/L	1.96 (1.62 to 2.34)	1.90 (1.56 to 2.29)	1.79 (1.45 to 2.20)
D-dimer-to-lymphocyte ratio	0.18 (0.13 to 0.26)	0.20 (0.14 to 0.30)	0.26 (0.17 to 0.38)

**Table 4:** Training configuration for the proposed model.

Hyperparameter	Value
Loss function	Focal loss (gamma = 2.0) plus entropy regularizer (lambda = 0.05)
Class imbalance handling	Focal loss and stratified folds; no class weighting
Post-hoc calibration	Temperature scaling fitted on validation folds
Optimizer	AdamW
Initial learning rate	1 × 10^−4^ image encoder; 3 × 10^−4^ tabular, microbiome, fusion
Learning rate schedule	Cosine annealing, minimum 1 × 10^−6^
Weight decay	1 × 10^−5^
Batch size	16
Maximum epochs	100
Early stopping	Patience 15 epochs, monitored on validation macro-AUC
Image input resolution	224 by 224 pixels
Fusion embedding dimension	256
Bottleneck tokens	4
Dropout rate	0.3
Modality dropout	0.15 per modality
Cross-validation	Five-fold, patient-level, class-stratified, repeated across five seeds

**Table 5:** Validation and external test performance of all configurations.

Model	Split	Accuracy	Sensitivity	Specificity	F1 Score	AUC (Macro)
Tabular only (gradient-boosted trees)	Validation	0.77 ± 0.04	0.74 ± 0.05	0.82 ± 0.03	0.73 ± 0.05	0.79 ± 0.04
Tabular only (gradient-boosted trees)	External	0.74 (0.66 to 0.81)	0.71 (0.63 to 0.78)	0.80 (0.73 to 0.86)	0.70 (0.62 to 0.77)	0.76 (0.68 to 0.83)
Image only	Validation	0.80 ± 0.05	0.78 ± 0.06	0.84 ± 0.04	0.77 ± 0.06	0.82 ± 0.05
Image only	External	0.79 (0.72 to 0.85)	0.77 (0.69 to 0.83)	0.83 (0.76 to 0.88)	0.75 (0.67 to 0.81)	0.81 (0.73 to 0.87)
Colposcopist impression	External	0.77 (0.69 to 0.83)	0.74 (0.66 to 0.81)	0.81 (0.74 to 0.87)	0.73 (0.65 to 0.80)	0.79 (0.71 to 0.85)
DLR plus microbiome	Validation	0.77 ± 0.03	0.74 ± 0.04	0.82 ± 0.03	0.73 ± 0.04	0.79 ± 0.03
DLR plus microbiome	External	0.76 (0.68 to 0.82)	0.73 (0.65 to 0.80)	0.81 (0.74 to 0.87)	0.72 (0.64 to 0.79)	0.78 (0.70 to 0.84)
Image plus DLR	Validation	0.85 ± 0.06	0.83 ± 0.07	0.88 ± 0.05	0.82 ± 0.07	0.87 ± 0.06
Image plus DLR	External	0.83 (0.76 to 0.88)	0.81 (0.74 to 0.87)	0.86 (0.79 to 0.91)	0.80 (0.73 to 0.86)	0.85 (0.78 to 0.90)
Image plus microbiome	Validation	0.84 ± 0.03	0.82 ± 0.04	0.88 ± 0.03	0.81 ± 0.04	0.86 ± 0.03
Image plus microbiome	External	0.84 (0.77 to 0.89)	0.81 (0.74 to 0.87)	0.88 (0.82 to 0.92)	0.81 (0.74 to 0.87)	0.86 (0.79 to 0.91)
Concatenation fusion	Validation	0.84 ± 0.05	0.82 ± 0.06	0.87 ± 0.04	0.81 ± 0.06	0.86 ± 0.05
Concatenation fusion	External	0.82 (0.75 to 0.87)	0.79 (0.72 to 0.85)	0.86 (0.79 to 0.91)	0.78 (0.70 to 0.84)	0.84 (0.77 to 0.89)
Cross-attention fusion	Validation	0.85 ± 0.04	0.83 ± 0.05	0.88 ± 0.03	0.82 ± 0.05	0.87 ± 0.04
Cross-attention fusion	External	0.84 (0.77 to 0.89)	0.82 (0.75 to 0.87)	0.87 (0.80 to 0.92)	0.81 (0.74 to 0.87)	0.86 (0.79 to 0.91)
Architecture 1, two-modality [11]	Validation	0.84 ± 0.04	0.81 ± 0.05	0.87 ± 0.03	0.81 ± 0.05	0.86 ± 0.04
Architecture 1, two-modality [11]	External	0.85 (0.78 to 0.90)	0.82 (0.75 to 0.87)	0.88 (0.82 to 0.92)	0.82 (0.75 to 0.87)	0.87 (0.80 to 0.92)
Architecture 1, three-modality [11]	Validation	0.85 ± 0.04	0.83 ± 0.05	0.88 ± 0.03	0.82 ± 0.05	0.87 ± 0.04
Architecture 1, three-modality [11]	External	0.86 (0.79 to 0.91)	0.84 (0.77 to 0.89)	0.89 (0.83 to 0.93)	0.83 (0.76 to 0.88)	0.88 (0.81 to 0.93)
Architecture 2, two-modality [12]	Validation	0.88 ± 0.07	0.86 ± 0.08	0.91 ± 0.05	0.85 ± 0.08	0.90 ± 0.07
Architecture 2, two-modality [12]	External	0.85 (0.78 to 0.90)	0.84 (0.77 to 0.89)	0.89 (0.83 to 0.93)	0.83 (0.76 to 0.88)	0.88 (0.81 to 0.93)
Architecture 2, three-modality [12]	Validation	0.88 ± 0.06	0.86 ± 0.07	0.91 ± 0.05	0.85 ± 0.07	0.90 ± 0.06
Architecture 2, three-modality [12]	External	0.86 (0.79 to 0.91)	0.83 (0.76 to 0.88)	0.89 (0.83 to 0.93)	0.82 (0.75 to 0.87)	0.88 (0.81 to 0.93)
Architecture 3, two-modality [14]	Validation	0.90 ± 0.06	0.88 ± 0.07	0.93 ± 0.05	0.87 ± 0.07	0.92 ± 0.06
Architecture 3, two-modality [14]	External	0.87 (0.80 to 0.92)	0.86 (0.79 to 0.91)	0.90 (0.84 to 0.94)	0.85 (0.78 to 0.90)	0.89 (0.82 to 0.93)
Architecture 3, three-modality [14]	Validation	0.89 ± 0.06	0.87 ± 0.07	0.92 ± 0.05	0.86 ± 0.07	0.91 ± 0.06
Architecture 3, three-modality [14]	External	0.86 (0.79 to 0.91)	0.84 (0.77 to 0.89)	0.89 (0.83 to 0.93)	0.83 (0.76 to 0.88)	0.88 (0.81 to 0.93)
Proposed TIM-Fusion	Validation	0.92 ± 0.02	0.90 ± 0.03	0.95 ± 0.02	0.89 ± 0.03	0.93 ± 0.02
Proposed TIM-Fusion	External	0.90 (0.84 to 0.94)	0.89 (0.83 to 0.93)	0.95 (0.90 to 0.98)	0.89 (0.83 to 0.93)	0.92 (0.86 to 0.96)

**Table 6:** Component ablation and missing-modality inference.

Configuration	AUC (Macro)	Difference from Full Model (95% CI)
Full TIM-Fusion	0.92 (0.86 to 0.96)	reference
Confidence weights fixed at one third	0.89 (0.82 to 0.93)	−0.03 (−0.08 to 0.02)
Entropy regularizer removed	0.90 (0.84 to 0.94)	−0.02 (−0.07 to 0.03)
Modality dropout removed	0.91 (0.85 to 0.95)	−0.01 (−0.05 to 0.03)
Bottleneck replaced by embedding concatenation	0.87 (0.80 to 0.92)	−0.05 (−0.10 to −0.01)
Bottleneck replaced and weights fixed at one third	0.84 (0.77 to 0.89)	−0.08 (−0.13 to −0.03)
Bottleneck tokens reduced to 1	0.90 (0.84 to 0.94)	−0.02 (−0.06 to 0.02)
Bottleneck tokens increased to 8	0.92 (0.86 to 0.96)	0.00 (−0.04 to 0.04)
Mixup removed from tabular branch	0.91 (0.85 to 0.95)	−0.01 (−0.05 to 0.04)
Inference with DLR withheld	0.88 (0.81 to 0.93)	−0.04 (−0.09 to 0.01)
Inference with microbiome withheld	0.86 (0.79 to 0.91)	−0.06 (−0.11 to −0.01)
Inference with image withheld	0.79 (0.71 to 0.85)	−0.13 (−0.20 to −0.07)
Inference with DLR withheld, age retained	0.89 (0.82 to 0.94)	−0.03 (−0.08 to 0.02)
Inference with age withheld, DLR retained	0.92 (0.86 to 0.96)	0.00 (−0.04 to 0.04)

**Table 7:** Per-class performance on the external test cohort.

Risk Category	*n*	TP	FP	FN	Sensitivity	Specificity	Precision	F1 Score	AUC
HPV-negative	56	52	2	4	0.93 (0.83 to 0.97)	0.98 (0.92 to 0.99)	0.96 (0.87 to 0.99)	0.95 (0.85 to 0.98)	0.95 (0.89 to 0.98)
HPV-positive without co-infection	58	52	6	6	0.90 (0.79 to 0.95)	0.93 (0.85 to 0.97)	0.90 (0.79 to 0.95)	0.90 (0.79 to 0.95)	0.93 (0.86 to 0.97)
HPV-positive with co-infection	26	22	6	4	0.85 (0.66 to 0.94)	0.95 (0.89 to 0.98)	0.79 (0.60 to 0.90)	0.81 (0.63 to 0.92)	0.87 (0.75 to 0.94)

**Table 8:** Secondary binary analysis, co-infection versus no co-infection within HPV-positive participants of the external cohort (*n* = 84, 26 events).

Metric	Value (95% CI)
True positives/false positives/false negatives/true negatives	22/5/4/53
ROC-AUC	0.86 (0.76 to 0.93)
Precision-recall AUC	0.72 (0.56 to 0.85)
Accuracy	0.89 (0.81 to 0.94)
Sensitivity	0.85 (0.66 to 0.94)
Specificity	0.91 (0.81 to 0.96)
Precision (PPV)	0.81 (0.63 to 0.92)
NPV	0.93 (0.83 to 0.97)
F1 score	0.83 (0.69 to 0.91)
Positive likelihood ratio	9.4 (4.0 to 22.3)
Negative likelihood ratio	0.16 (0.06 to 0.44)
Calibration intercept	0.05 (−0.30 to 0.40)
Calibration slope	0.88 (0.61 to 1.15)
Brier score	0.13

**Table 9:** Macro-AUC differences against each comparator.

Comparator	Macro-AUC Difference (95% CI)	*p* (Macro)	*p* (HPV−)	*p* (HPV+ No Co)	*p* (HPV+ Co)	Holm Outcome
Tabular only	0.16 (0.09 to 0.23)	<0.001	<0.001	<0.001	0.002	Significant
DLR plus microbiome	0.14 (0.08 to 0.20)	<0.001	<0.001	<0.001	0.004	Significant
Colposcopist impression (context only)	0.13 (0.06 to 0.20)	<0.001	<0.001	0.001	0.011	Not in family
Image only	0.11 (0.05 to 0.17)	<0.001	<0.001	<0.001	0.007	Significant
Concatenation fusion	0.08 (0.04 to 0.13)	<0.001	0.002	<0.001	0.019	Significant
Image plus DLR	0.07 (0.03 to 0.12)	0.001	0.009	0.003	0.052	Significant
Cross-attention fusion	0.06 (0.02 to 0.10)	0.002	0.004	0.006	0.031	Significant
Image plus microbiome	0.06 (0.02 to 0.11)	0.003	0.014	0.002	0.088	Significant
Architecture 1, two-modality [11]	0.05 (0.01 to 0.09)	0.004	0.006	0.017	0.043	Significant
Architecture 2, two-modality [12]	0.04 (0.01 to 0.08)	0.008	0.031	0.012	0.12	Significant
Architecture 1, three-modality [11]	0.04 (0.01 to 0.08)	0.011	0.023	0.009	0.076	Significant
Architecture 2, three-modality [12]	0.04 (0.004 to 0.077)	0.013	0.019	0.028	0.14	Significant
Architecture 3, three-modality [14]	0.04 (0.003 to 0.070)	0.018	0.041	0.022	0.11	Significant
Architecture 3, two-modality [14]	0.03 (0.002 to 0.061)	0.031	0.058	0.035	0.16	Significant

**Table 10:** External performance with and without MMUPHin batch correction (*n* = 140).

Metric	with Correction	Without Correction
Macro-AUC	0.92 (0.86 to 0.96)	0.91 (0.85 to 0.95)
Accuracy	0.90 (0.84 to 0.94)	0.89 (0.82 to 0.93)
AUC, HPV-negative	0.95 (0.89 to 0.98)	0.95 (0.89 to 0.98)
AUC, HPV-positive without co-infection	0.93 (0.86 to 0.97)	0.92 (0.85 to 0.96)
AUC, HPV-positive with co-infection	0.87 (0.75 to 0.94)	0.84 (0.71 to 0.92)
Sensitivity, co-infection class	0.85 (0.66 to 0.94)	0.81 (0.62 to 0.92)
Specificity, co-infection class	0.95 (0.89 to 0.98)	0.94 (0.88 to 0.97)
Precision, co-infection class	0.79 (0.60 to 0.90)	0.75 (0.56 to 0.88)
F1, co-infection class	0.81 (0.63 to 0.92)	0.78 (0.59 to 0.89)
Binary co-infection AUC within HPV-positive stratum	0.86 (0.76 to 0.93)	0.83 (0.72 to 0.91)
Calibration slope, co-infection class	0.93	0.87
Multiclass Brier score	0.07	0.08

**Table 11:** Calibration after temperature scaling.

Risk Category	Calibration Intercept (95% CI)	Calibration Slope (95% CI)
HPV-negative	0.02 (−0.10 to 0.14)	0.96 (0.81 to 1.11)
HPV-positive without co-infection	−0.03 (−0.16 to 0.10)	0.94 (0.78 to 1.10)
HPV-positive with co-infection	0.06 (−0.12 to 0.24)	0.90 (0.68 to 1.12)

**Table 12:** Subgroup performance on the external test cohort.

Subgroup	*n*	Co-Infection Events	AUC Type	AUC
Age below 35 years	74	14	Macro	0.91 (0.82 to 0.96)
Age 35 years and above	66	12	Macro	0.92 (0.82 to 0.97)
Lactobacillus-dominant CST	79	11	Macro	0.93 (0.83 to 0.97)
Non-Lactobacillus-dominant CST	61	15	Macro	0.90 (0.80 to 0.95)
No cervical intraepithelial neoplasia	57	10	Macro	0.90 (0.78 to 0.96)
CIN1	46	8	Macro	0.92 (0.79 to 0.97)
CIN2 or worse	37	8	Macro	0.93 (0.79 to 0.98)
HPV16 or HPV18	34	11	Binary	0.88 (0.66 to 0.97)
Other high-risk genotype	50	15	Binary	0.86 (0.68 to 0.95)

**Table 13:** Representative recent studies across the three literatures.

Study	Modality	Endpoint	Sample	Reported Metric	Validation
Zhuang et al. [5]	Blood ratio (NLR)	Prognosis, meta-analysis	38 studies, 10,246 patients	Pooled HR for overall survival 1.58 (1.44 to 1.74)	Meta-analysis
Chen JLY et al. [28]	Blood ratios (NLR, PNI)	Toxicity and prognosis	138 patients	HR for overall survival 3.83	Single-center
Yu et al. [29]	Blood ratios (NLR, PLR)	Chemoradiotherapy response	180 patients	AUC 0.848 (NLR)	Single-center
Tietie et al. [30]	Plasma D-dimer	Clinical utility	Not reported	Not reported	Single-center
Chen Q et al. [6]	Plasma D-dimer	Postoperative recurrence	888 patients	Adjusted OR 2.16 (1.28 to 3.62)	Single-center
Musa et al. [31]	Microbiome (16S, CST)	Precancer and cancer association	155 participants	Adjusted OR 2.72	Cross-sectional
Bautista et al. [7]	Microbiome	Narrative synthesis	Review	Not applicable	Not applicable
Ye and Qi [8]	Microbiome	Narrative synthesis	Review	Not applicable	Not applicable
Aquilina and Papagiannakis [11]	Colposcopy and referral data	Augmenting colposcopic impression	4946 images	AUC 0.75 image, 0.81 fused, 0.70 cross-camera	Internal 10-fold plus transfer test
Madathil et al. [12]	Colposcopy and clinical data	Multimodal classification	Not confirmed	AUC 0.999, accuracy 0.98	Internal only
Wang et al. [32]	Colposcopy	Lesion detection and localization	Multi-center	Accuracy 96.18%, AUC 0.99	Multi-center internal
Pacal and Kılıçarslan [33]	Cytology	Architecture benchmarking	Public dataset	Vision transformer ensembles above convolutional backbones	Public dataset split
Himabindu et al. [14]	Colposcopy	Swin ensemble classification	Not reported	Accuracy 99.44%	Internal only
TIM-Fusion (this study)	Blood, microbiome, and colposcopy	Co-infection classification	560 (420 development, 140 external)	Macro-AUC 0.92 (0.86 to 0.96)	Independent external center

## Data Availability

The data presented in this study are not publicly available because they comprise identifiable patient-level clinical, laboratory, microbiome, and colposcopic imaging records, and public deposition would be incompatible with the privacy conditions under which ethical approval and the consent waiver were granted. De-identified data supporting the reported results are available from the corresponding author (L.S.) on reasonable request, subject to approval by the institutional review boards of the participating centers and a data transfer agreement. The colposcopic images cannot be shared in any form.

## Supplementary Materials

The following supporting information can be downloaded at: https://www.mdpi.com/article/10.3390/diagnostics16182992/s1, Table S1: External confusion matrix for the proposed model; Table S2: Per-organism composition of the co-infection category; Figure S1: Training and validation curves for every configuration.

## Abbreviations

The following abbreviations are used in this manuscript:

ASV	Amplicon sequence variant
AUC	Area under the receiver operating characteristic curve
CI	Confidence interval
CIN	Cervical intraepithelial neoplasia
CLR	Centered log-ratio
CST	Community state type
DLR	D-dimer-to-lymphocyte ratio
FEU	Fibrinogen-equivalent units
FT-Transformer	Feature-tokenizer transformer
HPV	Human papillomavirus
HR	Hazard ratio
IQR	Interquartile range
MoCo	Momentum contrast
NLR	Neutrophil-to-lymphocyte ratio
OR	Odds ratio
PCR	Polymerase chain reaction
PLR	Platelet-to-lymphocyte ratio
PNI	Prognostic nutritional index
ROC	Receiver operating characteristic
SHAP	SHapley Additive exPlanations
SMD	Standardized mean difference
TIM-Fusion	Tri-modal Integration Model Fusion
TRIPOD+AI	Transparent Reporting of a multivariable prediction model for Individual Prognosis Or Diagnosis, artificial intelligence extension
t-SNE	t-distributed stochastic neighbor embedding
