# Supplementary material for: Colposcopic Imaging Carries Most of the Signal: Externally Validated Tri-Modal Integration of Imaging, Vaginal Microbiome, and Coagulation-Immune Data in HPV-Associated Co-Infection
Source: Diagnostics (Basel). 2026 Sep 16;16(18):2992. doi: 10.3390/diagnostics16182992 (PMC13605049; doi:10.3390/diagnostics16182992)
Supplement: Supplementary file 1 [file diagnostics-16-02992-s001.zip › diagnostics-4529092-supplementary.pdf]

Supplementary Table S1. External confusion matrix for the proposed model

True category	Predicted HPV negative	Predicted HPV positive without co-infection	Predicted HPV positive with co-infection	Total
HPV negative	52	3	1	56
HPV positive without co-infection	1	52	5	58
HPV positive with co-infection	1	3	22	26
Total predicted	54	58	28	140

Supplementary Table S2. Per-organism composition of the co-infection category

Organism	Detected by 16S profiling	Development cohort (76 cases)	External cohort (26 cases)
Chlamydia trachomatis	Intracellular, inconsistently amplified	31	11
Trichomonas vaginalis	Absent, a protozoan	22	7
Mycoplasma genitalium	Genus-resolvable, masked a priori	18	6
Neisseria gonorrhoeae	Usually below detection at screening read depth	9	3
Total detections		80	27
Participants positive for two organisms		4	1

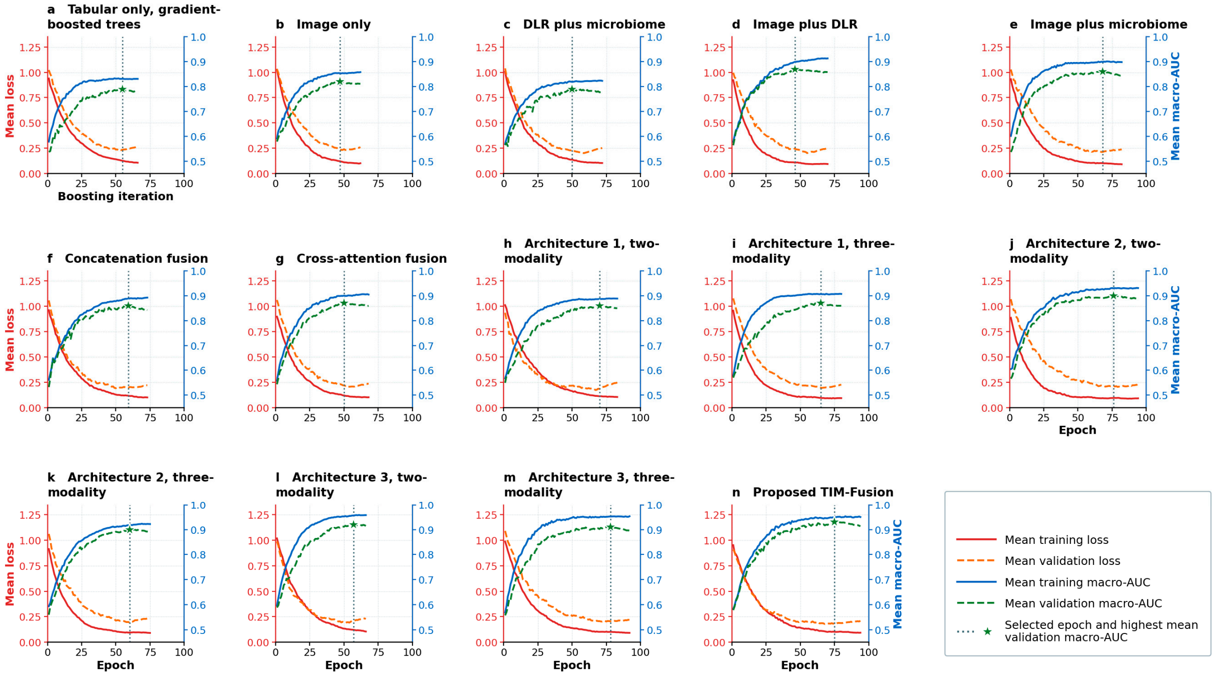

Supplementary Figure S1. Training and validation curves for every configuration. Panels a to n correspond to the fourteen configurations of Table 5, in the same order. Solid red and dashed orange give mean training and validation loss on the left axis; solid blue and dashed green give mean training and validation macro-AUC on the right. Each curve is the mean over the 25 fits of the configuration, five folds by five seeds, computed at each epoch index over the fits still training at that point; no interpolation or padding was used, so the curves end where the longest fits end. The dotted vertical line and the star together mark the selected epoch, the one at which mean validation macro-AUC was highest and at which weights were carried forward under early stopping with a patience of 15 epochs. For panel a the abscissa is the boosting iteration rather than the epoch. Validation curves are computed on the held-out fold and use no external data.

.
